# Administration of Vaccines in Dairy Sheep and Goat Farms: Patterns of Vaccination, Associations with Health and Production Parameters, Predictors

**DOI:** 10.3390/vaccines10091372

**Published:** 2022-08-23

**Authors:** Daphne T. Lianou, Charalambia K. Michael, Efthymia Petinaki, Vasia S. Mavrogianni, George C. Fthenakis

**Affiliations:** 1 Veterinary Faculty, University of Thessaly, 43100 Karditsa, Greece; 2 University Hospital of Larissa, 41110 Larissa, Greece

**Keywords:** abortion, enterotoxaemia, farmer, goat, health management, mastitis, paratuberculosis, pneumonia, sheep, vaccine

## Abstract

This paper reports findings regarding patterns of vaccine usage in sheep and goat farms, in 325 sheep flocks and 119 goat herds throughout Greece. The objectives of the study were (a) to describe the patterns of vaccine administration in small ruminant farms and (b) to highlight factors that were associated with vaccinations in the farms. Vaccination against brucellosis was performed in all farms into the study. Among optional vaccinations, anti-clostridial vaccination was most frequently performed (in 97.8% of farms), followed by vaccination against contagious agalactia, (56.5% of farms), pneumonia (41.2%), chlamydial abortion (38.1%), staphylococcal mastitis (36.0%), and paratuberculosis (9.5%). Vaccinations against pneumonia and staphylococcal mastitis were performed more frequently in sheep flocks, whilst vaccinations against paratuberculosis were performed more frequently in goat herds. On average, 2.8 and 2.7 optional vaccinations (i.e., additionally to vaccination against brucellosis) were performed in sheep and goat farms, respectively. The increased number of vaccines administered was associated with a higher average milk production in the respective farms. There was an association of vaccination against staphylococcal mastitis with a reduced recovery of staphylococci from the bulk-tank raw milk. In multivariable analyses, significant associations of the administration of the various optional vaccines were seen with 15 variables, 11 related to health management practices and 4 related to the demographic characteristics of farmers; the collaboration with a veterinarian, the daily number of milking sessions, and the period spent daily by the farmer at the farm premises were each associated with the administration of vaccines against three infections.

## 1. Introduction

Vaccinations have had significant beneficial effects on small ruminant health and production and are paramount components of health management programs applied in sheep and goat farms [1]. Usually, the implementation of correct vaccination programs in flocks and herds should be based on the evaluation of various parametres; some of these are the production type, the environmental and climatological factors in the area, the infections prevailing in the farm, the infrastructure of the farm and the human resources, etc. The correct administration of vaccines and the application of relevant vaccination programs is important for ensuring the maintenance of health and high standards of animal welfare in the farms. There is also a public health significance, as vaccines often prevent infections with zoonotic importance (e.g., staphylococcal mastitis and *Toxoplasma gondii* infection). Thus, knowledge of the patterns of vaccine administration in livestock farms can be employed to ensure that the vaccines are administered properly and that they provide a high protection in the target animals.

There is a paucity of relevant information internationally about vaccination programs clinically applied in sheep and goat farms. Most relevant studies have focused on the importance of vaccination against brucellosis [2,3,4], a disease with a high public health significance, for which vaccination programs are designed and implemented at a national basis by the official veterinary services [5]. Another infection for which several related works have been published is peste des petits ruminants, a significant infection of sheep and goats, for which control programs are carried out by veterinary services [6,7]. Fewer reports have dealt with vaccination programs in small ruminant infections, control plans for which are designed mostly at the farm level and relevant decisions are taken by farmers and the technical consultants, e.g., bacterial mastitis [8], bacterial pneumonia [9], contagious agalactia [10], or paratuberculosis [11].

In small ruminants, the CORE vaccination is internationally the anti-clostridial infection vaccination [12]. However, local situations in the various areas of the world may set additional vaccination requirements, driven by morbidity by specific infections or production requirements. For example, in the United Kingdom, vaccinations against causes of abortion have been important and prioritised in sheep flocks [13], whilst in New Zealand, vaccination against foot-rot has been traditionally widely performed [14].

Dairy sheep and goat farming is the leading sector of the agricultural industry in Greece and has a large and important milk production. There are approximately 8.4 million sheep and 3.6 million goats in Greece, which represent 6.5% and 22.0% of the respective animal numbers in Europe. Milk production from these animals in total covers approximately 15% of the entire European milk production from small ruminants [15], which makes Greece a significant milk producer.

Despite the importance of small ruminant farming for the food production sector in Greece, the patterns of vaccine administration in sheep and goat farms in the country have not been investigated and described. This paper reports findings regarding patterns of vaccine usage in sheep and goat farms, as found in a detailed countrywide investigation carried out in 325 sheep flocks and 119 goat herds around the country. The objectives of the study were (a) to describe the patterns of vaccine administration in sheep and goat farms and (b) to describe factors that were associated with vaccinations in the farms.

## 2. Materials and Methods

### 2.1. Small Ruminant Farms and Interviews of Farmers

**Figure 1:**
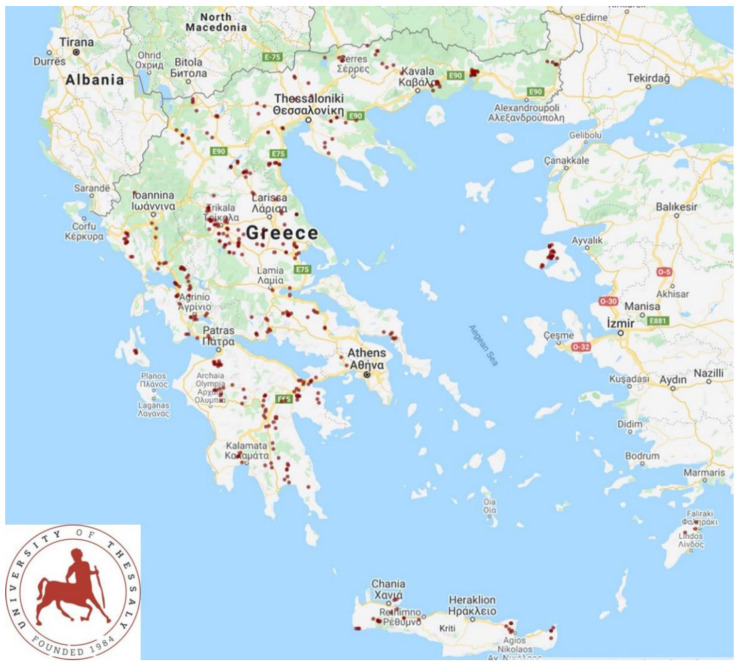
Map presenting locations of the 444 small ruminant farms throughout Greece, which were visited to record details on vaccination schedules and procedures.

We performed a cross-sectional study (April 2019–June 2020) in 325 sheep flocks and 119 goat herds. The study expanded in all the 13 administrative regions of Greece (Figure 1). Details and the protocol for inclusion of flocks and herds in the study have been presented before [16]. During the study, we visited all the flocks and herds, in the company of local veterinarians, practicing in small ruminant health management [16]; this collaboration involved 47 veterinarians.

At the start of each visit to the farms, the accompanying veterinarians made the introductions. The objectives and the details of the study were presented to and discussed with the farmers by the senior author (G.C.F.), who then introduced the interviewer (author D.T.L.).

The interview was performed using a structured detailed questionnaire. In a preliminary study, this questionnaire had been tested for validity of the content [16]. In a previous publication, we have presented all the minute details of the questionnaire employed and the interview procedures performed [16].

### 2.2. Milk Samples and Laboratory Examinations

After completion of the interview, two milk samples (20 mL each) were collected directly from the bulk-tank of the farm by means of aseptic procedures, with the aim to perform bacteriological examinations in the samples. These were transported to the laboratory by the investigators themselves, in cool conditions (0.0 to 4.0 °C), by car, airplane, and/or boat, according to the locality of each farm.

Within 24 h after sample collection, bacteriological techniques were employed for staphylococcal recovery and identification. Each sample was divided in two equal sub-samples, which were processed separately to increase sensitivity of the procedure. Full details of the techniques and procedures employed for bacterial recovery and identification have been described before [17,18]. Detection of confirmed staphylococcal colonies on at least one agar plate from subsamples obtained from each bult-tank milk was deemed to indicate presence of staphylococci in the bulk-tank milk. Definite staphylococcal identification was made by means of matrix-assisted laser desorption/ionization time-of-flight mass spectrometry (VITEK MS; BioMerieux, Marcy-l’-Étoile, France) [19,20].

Biofilm formation by staphylococcal isolates obtained, was carried out by combining the results of two tests, specifically culture appearance on Congo Red agar plates and results of microplate adhesion test, as presented before by Vasileiou et al. [21]. Staphylococcal isolates were finally classified as biofilm-forming (‘slime-producing’) or non-biofilm-forming (‘non-slime-producing’).

### 2.3. Data Management and Analysis

Data were entered into Microsoft Excel (Microsoft Corporation, Redmond, WA, USA) and analysed using SPSS v. 21 (IBM Analytics, Armonk, NY, USA).

Application of vaccinations against the following ten (10) infections in the farms was assessed: brucellosis (infection by *Brucella melitensis*) (compulsory vaccination in the mainland of the country and compulsory non-vaccination in the islands (bar in the island of Lesvos, where vaccination is compulsory)), chlamydial abortion, clostridial infections, contagious agalactia, contagious ecthyma, foot-rot, paratuberculosis, pneumonia, staphylococcal mastitis, and *Toxoplasma gondii* abortion (optional vaccinations). The specific products administered in each farm and the vaccinations schedules employed were also considered, if provided by farmers. The above infections are important problems and significant causes of deaths and production losses in small ruminant farms and their control and prevention encompasses use of vaccinations. Initially, we performed basic descriptive analyses, and we obtained exact binomial confidence intervals (CIs). Results in sheep and goat farms were analysed and presented separately.

The total number of optional vaccinations performed in a farm was calculated and the potential association with average milk production per animal in the respective farms was evaluated. The potential associations of vaccination against contagious agalactia or staphylococcal mastitis with the average milk quantity per female animal in a farm and the isolation of staphylococci or biofilm-forming staphylococci were assessed by using analysis of variance or Pearson’s chi-squared test, as appropriate.

The total number of vaccinations applied in farms against two infections (clostridial infections and pneumonia) that might lead in mortality of newborns was associated with the proportion of lambs or kids that were sold for slaughter compared to the total number born in the farms, by using Pearson’s chi-squared test. The total number of vaccines applied in farms against five infections (clostridial infections, contagious agalactia, paratuberculosis, pneumonia, staphylococcal mastitis) that might lead in mortality of adult animals was associated with the annual incidence of mortality of adult animals in the farms, by using analysis of correlation.

In total, eight outcomes were considered, which referred to optional vaccinations: ‘vaccination against xxxx’, where xxxx = chlamydial abortion, clostridial infections, contagious agalactia, contagious ecthyma, footrot, paratuberculosis, pneumonia, or staphylococcal mastitis. Vaccination against brucellosis was not included, as this was fully regulated by the relevant decrees and official procedures. The variables evaluated for potential associations with the above outcomes are in Table S1; we obtained outcomes for these variables directly, i.e., from the answers of farmers during the interview performed at the farm, or, alternatively, we calculated outcomes based on the answers of the farmers. For each of these variables, categories were created according to the answers of the farmers; for the analysis of the potential associations with breeds of animals in the farms, breeds were clustered into crossbreeds, imported breeds, and Greek (local) breeds. Separate analyses were performed for sheep and for goat farms. Exact binomial CIs were obtained. Initially, for each of the above right outcomes, univariable analysis was performed using Pearson’s chi-squared test in cross-tabulation and with simple logistic regression. Subsequently, again separately for each outcome, a multivariable model was constructed for each outcome. We offered to this model variables, which were found with of *p* < 0.2 in the preceding univariable analyses. Then, progressively, we removed variables from the model by using backward elimination. The likelihood ratio test was performed to assess *p*-value of each variable; among those found with *p* > 0.2, the one with the largest *p* was removed from the model. We repeated this procedure until we could not remove any value from the model, with *p* > 0.2. The variables included in the final multivariable models constructed for each outcome are detailed in Table S2.

The outcome ‘*total number of optional vaccines administered*’ was considered. Variables, which were included in at least one of the final multivariable models during assessments of the eight outcomes described hereabove (*n* = 16 for sheep and *n* = 16 for goat farms, details in Table S2), were assessed for possible associations with the present outcome. Separate analysis was performed for sheep and goat farms. The procedures for univariable and multivariable analyses, described in detail hereabove, have also been employed. The variables included in the final multivariable models are detailed in Table S2.

In all analyses, statistical significance was defined at *p* < 0.05.

## 3. Results

### 3.1. Descriptive Findings and Programs of Vaccinations in the Farms

Vaccination against brucellosis, which is compulsory in the mainland of the country, as part of the national campaign for eradication of the disease and prevention of human infections, was performed in all farms into the study. The Rev-1 vaccine was used in all farms. In all cases (100% of sheep and goat farms), the vaccine was administered in female animals maintained for replacement.

Among optional vaccinations, anti-clostridial vaccination was the one most frequently performed, in 434 (97.8%) farms, followed by vaccination against contagious agalactia, in 251 (56.5%) farms. In contrast, vaccination against abortion caused by *T. gondii* was not performed in any farm (0.0%) (Table 1).

Vaccinations against pneumonia and staphylococcal mastitis were performed more frequently in sheep flocks than in goat herds: 44.3% and 38.8%, respectively, versus 32.8% and 28.6%, respectively (*p* = 0.028 and 0.047, respectively). In contrast, vaccinations against paratuberculosis were performed more frequently in goat herds than in sheep flocks: 26.1% versus 3.4% (*p* < 0.0001). For the other vaccinations, the proportions of sheep and goat farms in which the respective vaccines were administered did not differ significantly (*p* > 0.15) (Table 1).

On average, 2.8 ± 0.1 optional vaccinations were performed in sheep flocks and 2.7 ± 0.1 in goat farms on average (*p* = 0.53). In 111 (25.0%) farms, at least three, and in 78 (17.6%) at least four different optional vaccinations were performed. In six (1.4%) farms, no optional vaccinations were performed.

The specific products that were employed in the optional vaccinations are detailed in Table S3. Several farmers chose to use more than one commercial products for the same optional vaccination. Specifically, for contagious agalactia 8 (4.3%) sheep and 1 (1.5%) goat farmers, for pneumonia 3 (2.1%) sheep and 2 (5.1%) goat farmers and for staphylococcal mastitis 1 (0.8%) sheep and 1 (3.9%) goat farmers used two different commercial products; for clostridial infections, 58 (19.7%) sheep and 25 (23.1%) goat farmers used two, 6 (2.0%) and 7 (6.5%) used three and 2 (0.7%) and 1 (0.9%), respectively, used four different commercial products. There was a significant difference between the frequency of commercial products used in sheep flocks and goat herds against clostridial infections (*p* = 0.026), but not for the frequency of commercial products used against other infections (*p* > 0.16 for all other comparisons).

For optional vaccinations, details of vaccination schedules applied in the farms were provided by > 7 farmers for clostridial infections (*n* = 416), contagious agalactia (*n* = 66), and staphylococcal mastitis (*n* = 134). These vaccines were administered most frequently to pregnant females. The assessment of these results did not show a difference in vaccination schedules applied between sheep and goat farms. Details are in Table 2.

### 3.2. Associations with Parameters Related to Health and Production in the Farms

**Figure 2:**
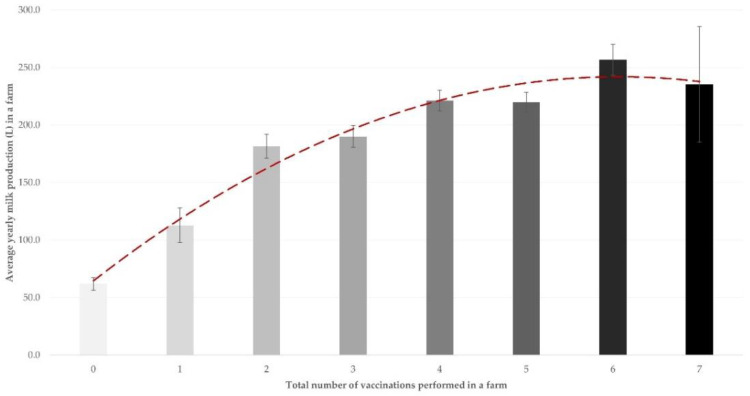
Association between the sum of optional vaccinations performed in a farm and the average milk production per animal, as found in 444 small ruminant farms in Greece (bars show standard error of the mean, red dashed line is respective trendline).

There was a correlation between the sum of optional vaccines administered in a farm and the average milk production per animal in the respective farms, for sheep flocks (*r* = 0.343, *p* < 0.0001) and goat herds (*r* = 0.172, *p* = 0.031). The increased number of vaccines administered was clearly associated with a higher average yearly milk production in the respective farms (*p* < 0.0001) (Figure 2, Table S4).

In sheep flocks, vaccination against contagious agalactia and/or staphylococcal mastitis was clearly associated with higher average milk production (*p* < 0.0001), whilst in goat herds, only a tendency for such an association was seen (*p* = 0.08) (Table 3). For both sheep and goat farms, no difference was seen in milk production between farms, in which either of these two vaccines was used (*p* > 0.75).

**Figure 3:**
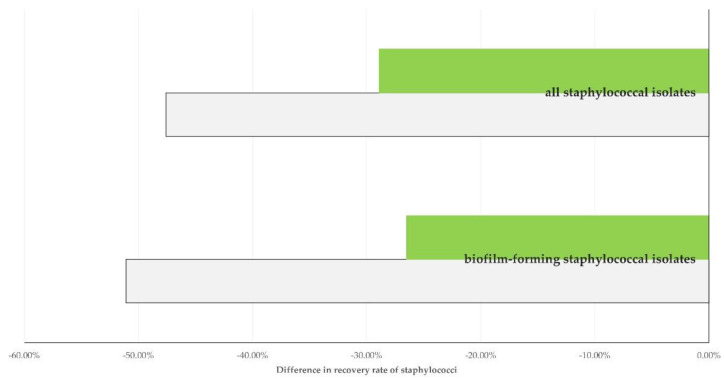
Difference in isolation rate of staphylococci from farm bulk-tank milk from sheep flocks (green) or goat herds (grey) in Greece, in which vaccination against staphylococcal mastitis was applied, compared to farms, in which no such vaccination was performed.

With regard to parameters related to milk quality, there was a clear association of vaccination against staphylococcal mastitis with a reduced recovery of staphylococci from the farm bulk-tank raw milk. Among the 126 sheep flocks in which vaccination against mastitis was applied, staphylococci and biofilm-forming staphylococci were recovered from the farm bulk-tank milk samples from 64 (50.8%) and 47 (37.3%) flocks, respectively, whilst of the 199 unvaccinated flocks, they were recovered from samples from 142 (71.4%) (*p* = 0.0002) and 101 (50.8%) flocks (*p* = 0.018), respectively. Of the 34 goat herds in which vaccination against mastitis was applied, they were recovered from bulk-tank milk samples from 13 (38.2%) and 9 (26.5%) herds, respectively, whilst of the 85 unvaccinated herds, they were recovered from samples from 62 (72.9%) (*p* = 0.0004) and 46 (54.2%) herds (*p* = 0.006), respectively (Figure 3).

**Figure 4:**
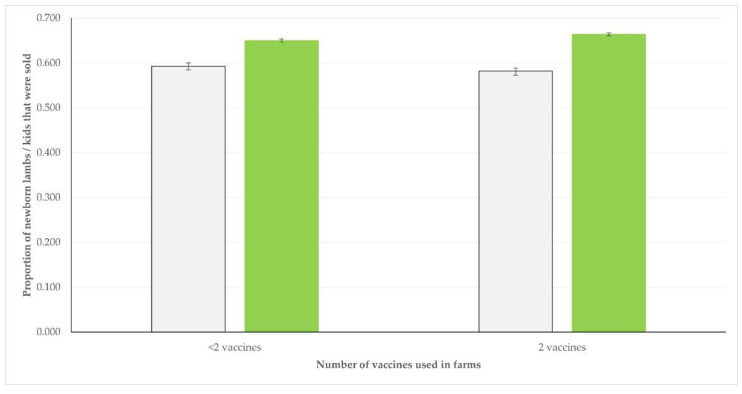
Proportion of newborns that were sold of those born, in sheep flocks (green) or goat herds (grey) in Greece, according to the total number of vaccines against two infections that might lead in death of affected newborns, which were used in the farms (bars show 95% CIs).

In relation to vaccinations against infections that might lead to the mortality of newborns (clostridial infections, pneumonia), in sheep farms, there was a clear association between vaccinations performed against those infections and the proportion of newborn lambs that were sold: 66.4% (95% CIs: 66.0–66.8%) in flocks where both vaccines were used versus 65.0% (95% CIs: 64.6–65.4%) in flocks where no vaccination or only one vaccine was used (*p* < 0.0001). However, no such difference was seen in goat herds: 58.2% (95% Cis: 57.3–59.1%) versus 59.3% (95% Cis: 58.6–60.0%) (*p* = 0.09) (Figure 4).

**Figure 5:**
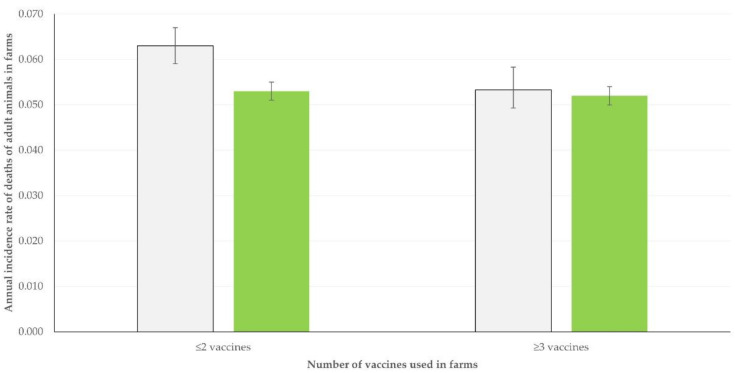
Incidence rate of mortality of adult animals in sheep flocks (green) or goat herds (grey) in Greece, in accord with the total number of vaccines against infections that might lead in death of affected adult animals, which were used in the farms (bars show 95% CIs).

In contrast, in relation to vaccinations against infections that might lead to the mortality of adult animals (clostridial infections, contagious agalactia, paratuberculosis, pneumonia, staphylococcal mastitis), in goat farms, there was a clear association between the total number of vaccines administered against those infections and the annual incidence rate of mortality of adult animals: 6.30% (95% CIs: 5.94–6.68%) in herds where up to two vaccines were used versus 5.33% (95% CIs: 4.94–5.75%) in herds where at least three vaccines were used (*p* = 0.0007). However, no such difference was seen in sheep flocks: 5.32% (95% CIs: 5.14–5.50%) versus 5.15% (95% CIs: 4.96–5.37%), respectively (*p* = 0.23) (Figure 5).

### 3.3. Predictors

The full results of the univariable analyses performed for the assessment of possible associations of predictors with optional vaccination against chlamydial abortion, clostridial infections, contagious agalactia, contagious ecthyma, foot-rot, paratuberculosis, pneumonia, and staphylococcal mastitis, are in Tables S5–S12. The results are presented separately for sheep flocks and goat herds.

Overall, in the multivariable analyses, we found significance for seven outcomes in relation to sheep flocks and for five outcomes in relation to goat herds. Of the 15 variables that were found cumulatively with a significant association with the totality of the various outcomes assessed (12 in sheep flocks and 10 in goat herds), 11 were related to health management practices performed in the farms and four to the demographic characteristics of farmers (8 and 4, respectively, in sheep flocks and 8 and 2, respectively, in goat herds). Specifically, the collaboration with a veterinarian, the daily number of milking sessions, and the period spent daily by the farmer at the farm premises were each associated with three outcomes; another six variables (four health management practices: age of newborns when taken away from dam, average age of culling female animals, management system applied in the farm, and the use of laboratory diagnostic examinations in milk samples, and two demographic characteristics of farmers: family tradition in farming and farmer’s general education) were each associated with two outcomes. The findings of the multivariable analyses are summarized in Table 4, whilst the detailed results of these analyses with odds ratio(s) for each significant predictor are in Table A1, Table A2, Table A3, Table A4, Table A5, Table A6, Table A7 and Table A8. The results are presented separately for sheep flocks and goat herds.

Specifically with regard to the breed of animals in the farms, in sheep flocks, associations with vaccinations were found for contagious agalactia (*p* = 0.002) (Table S7) and staphylococcal mastitis (*p* = 0.019) (Table S12). In goat herds, associations with vaccinations were found for chlamydial abortion (*p* = 0.013) (Table S5) and contagious agalactia (*p* = 0.004) (Table S7). In these four cases, vaccinations were carried out more frequently in farms with imported breeds: in 68.1% and 47.5%, respectively, of sheep flocks and in 46.7% and 64.1%, respectively, of goat herds (Tables S5, S7 and S12).

### 3.4. Total Number of Vaccinations Performed in Farms

The full results of the univariable analyses performed for the assessment of possible associations of predictors with the sum of optional vaccinations are in Table S13. In the multivariable analyses, in sheep flocks, seven variables were found to be significantly associated with the total number of optional vaccines administered (*p* < 0.03), whilst in goat herds, only one variable was found with a significance (*p* = 0.036). The results of the analyses are shown in Table 5.

Specifically with regard to the breed of animals in the farms, there was clear evidence, for both sheep and goat farms, that a significantly higher number of optional vaccinations were performed in farms with imported breeds of animals (*p* < 0.01) (Table 6).

## 4. Discussion

### 4.1. Preamble

Sheep and goat farming is currently the stronger branch of animal production industry in Greece, making approximately 18% of the total income of the primary sector income [22]. In over 98% of farms, sheep and goat farming in Greece refers to dairy production. In fact, in the country, milk production from sheep and goats exceeds by far the milk production from cattle [23]. The correct application of vaccination programs will contribute greatly to an improved health status and increased production in small ruminant farms [1], although the generally low value of individual sheep and goats imposes some constraints in vaccinating them [12].

In this work, we studied the patterns of vaccination programs applied in 444 small ruminant farms (325 sheep flocks and 119 goat herds) during an extensive countrywide investigation. During the study, we also assessed potential associations with health-related procedures and practices and the human resources available on the farms. We included farms from all the administrative regions of Greece into the study, by means of which, situations and conditions prevailing in all areas of the country were considered and thus factors of regional importance did not play a predominant role. As far as we are aware, this was the largest sample size employed ever, as reported in the international literature, to investigate the present topic. Whilst the significance of applying correct vaccinations schemes is widely recognised, there is a paucity of detailed information and, moreover, data regarding the patterns of usage of compulsory vaccines, e.g., against brucellosis, have been mainly published.

According to data sourced from the Hellenic Milk Board [24], the farms included in this study refer to approximately 1% of the total number of sheep and goat farms in Greece. Farms were included in the study on a convenience basis, but the approach employed guaranteed that farmers would accept the visit, whilst the presence of an accompanying local veterinarian contributed to minimising suspiciousness and distrust from their part, which consequently led to a relaxed interview. During the study, we used consistent methodologies and ensured that the interviews were always performed by the same investigator (author D.T.L.).

Furthermore, our approach allowed the inclusion of flocks and herds with farmers genuinely willing to participate in the study and to provide thoughtful and correct answers. A degree of stratification was employed in the selection of farms, as the flocks and herds visited were located in all 13 administrations of the country. Whilst the general limitations of questionnaire surveys applied in this work (e.g., unconscientious responses by farmers and differences in understanding and interpretation of the question) [25], we made our utmost to decrease any possible adverse effects in the study; for example, queries of the respondents were answered immediately by the interviewer (author D.T.L.) [16], and, at the same time, the principal author (author G.C.F.) discussed some of the answers of the interviewees with the veterinarians accompanying in the farms, with the objective to verify the accuracy of responses provided [16].

### 4.2. Vaccination Patterns

First, it is paramount to comment that the compulsory vaccination against brucellosis was reportedly performed in all the farms into the study. In the 10 last years, the Ministry of Rural Development and Food of the country has allocated significant resources for correct implementation of this vaccination in the country, with the aim to minimise cases of the infection in people [26]. As a result, cases of brucellosis in people in the country have been decreasing [27]. These reports are in line with the current results, which indicate the wide dissemination of the procedure and full implementation of vaccination, as the incidence of brucellosis in people in an area fluctuates in accord with the success of the vaccination program of small ruminants [28,29].

With regard to optional vaccinations, anti-clostridial vaccination, which is the CORE vaccination in small ruminants [12], was, as expected, the one more often performed. Although it is recommended that this vaccination is performed twice yearly [12,30], only a small minority of farmers applied that.

Other vaccines used in several of the farms visited (over 35% of them) were the vaccines against contagious agalactia, pneumonia, chlamydial abortion, and staphylococcal mastitis. These infections are prevalent clinical problems of small ruminants in Greece [31,32,33,34,35] and thus, vaccination is among the measures applied for their control. The increased rate of vaccination against contagious agalactia and staphylococcal mastitis reflects the orientation for dairy production of the small ruminant industry in Greece. However, it was surprising to see only 36% of the farmers vaccinating against staphylococcal mastitis, given that, during the present investigation, 59.9% of the same farmers reported that they considered mastitis as the most significant health problem of adult animals in their farms (unpublished data).

The significantly higher vaccination rate against paratuberculosis among goat herds than sheep flocks reflects the higher susceptibility of goats than sheep to the causal pathogen [36,37]. The infrequent vaccination against contagious ecthyma is the consequence of the requirement for a specific import licence on a farm-by-farm basis, in accord with the incidence of the infection in the farms. Finally, despite the presence of *T. gondii* infections in small ruminants in Greece and the widespread cases of abortions caused by this pathogen [38,39], also with zoonotic implication [40], issues in the marketing of the relevant commercial product have likely impeded the use of the vaccine in the farms.

### 4.3. Associations with Health and Production in the Farms

Vaccinations were clearly associated with improved health and production outcomes. First, the total number of vaccines was found to be associated with a higher milk production in the respective farms. This can indicate a direct beneficial effect of the vaccinations in the health of the animals in the farm, by protecting against more infections, which has resulted in a higher milk production. However, it is more likely that it reflects farmers generally more attentive to their business and applying improved management in all its facets, including nutrition, infrastructure (e.g., machine milking), number of daily milkings, breed of animals, characteristics of farmers (e.g., experience), etc., as well as vaccinations; hence, the higher milk production is the consequence of the high level of management, part of which is the higher number of vaccinations. Thus, the association between the increased number of vaccinations and the higher milk production is more likely a spurious one, rather than one indicating causation.

Vaccination against staphylococcal mastitis was found to be associated with the reduced recovery of staphylococci and biofilm-forming staphylococci in the bulk-tank milk. However, in previous studies, no beneficial effects have been found in quality parameters of the milk, specifically, somatic cell counts, total bacterial counts, and chemical composition [19,20,41]. Vaccines licenced for the prevention of mastitis of sheep and goats offer protection against mastitis caused by staphylococci only [8], whilst mastitis can be caused by a variety of pathogens (including *Mannheimia haemolytica*, streptococci, etc.,) [32,42], against which these vaccines are ineffective. In addition, vaccines against staphylococcal mastitis offer a reduction in infection by staphylococcal strains, hence some shedding in milk has been reported in vaccinated animals [43,44]. The present findings can be aligned to previous studies, where we reported a reduced number of staphylococcal recovery from milking system teatcups in flocks and herds vaccinated against staphylococcal mastitis, compared to unvaccinated ones [45].

### 4.4. Factors Associated with the Vaccinations

Most of the variables identified with association to the administration of a vaccine are related to factors that facilitate the administration of the vaccines. For example, the increased daily presence of farmers in their business allows more time available for performing essential tasks, among them vaccinations; in another context, in farms where three milking sessions are applied daily, animals are gathered in the milking parlour more frequently and thus can be vaccinated more easily. The management system applied in farms can be related to both the feasibility of administration of vaccines (easier in intensively or semi-intensively managed farms, where animals are maintained indoors), and also to the increased infectivity of some pathogens (also easier in intensively or semi-intensively managed farms, for example, *Mycoplasma agalactiae* is transmitted through the milking machine teatcups during milking [46]).

Other variables found to be associated with the vaccinations can refer to various facets of health management in farms. For example, the average age of culling female animals can be related to the value of female animals; an early culling age of ewes does reflect a shorter production life-span of these animals, which thus need to be protected to prevent infections during that period. Moreover, the use of laboratory diagnostic examinations in samples of milk (found to be associated with the administration of the two vaccines related to mammary infections, contagious agalactia and staphylococcal mastitis) may indicate the interest of farmers το maximise and monitor milk production in their farms.

The regular collaboration with a veterinarian facilitates the design and implementation of health management plans, part of which are the vaccinations [12]. Moreover, in such cases, vaccination schedules can be applied correctly rather than in a badly organised manner, whilst veterinarians will also supervise the correct administration of vaccines (given that in some cases, these products include attenuated microbial strains, which can cause zoonotic infections, e.g., brucellosis, chlamydial abortion, and contagious ecthyma) and can monitor animals for post-administration adverse reactions.

The age of the farmer emerged as the most significant variable for the total number of vaccinations performed. In this respect, it is worth citing that Tauer [47] reported that the productivity of farms owned by farmers older than 45 years decreased progressively with the age of the farmers. The reduced number of vaccinations performed in farms run by people aged over 50 years can contribute to such an outcome. It has in fact been reported that New Zealand sheep farmers aged over 50 years used fewer health management tools than their younger colleagues, and moreover, these farmers were also omitting even basic procedures, including vaccinations against clostridial infection in their flocks [48], findings that have a similarity with results of the present study.

The breed of animals was found to be associated with the administration of some optional vaccines. It has been well-established that the animal breed can play a role in an increased susceptibility or resistance of animals to some infections, for example mastitis [49,50], the findings (i.e., the lack of differences in some evaluations or the differences in other ones) may reflect this aspect, i.e., farmers who understand that their animals are more susceptible to some infections, perform vaccinations aiming to increase their protection. The finding of the increased proportion of vaccinated farms among those with imported breeds, lends some support to this hypothesis, as local breeds would likely be more resilient to infections, as better adopted to local conditions.

## 5. Conclusions

The study explored the vaccination patterns in small ruminant farms. Vaccinations have contributed to controlling many major infectious diseases, despite the misinformation and the unsubstantiated resistance to their use. The correct implementation of vaccination programs, based on sound scientific principles and full compliance with established regulations and policies, are important for the improvement of the welfare of farm animals. Although most sheep and goat farms function at a low technological level, these species are important livestock animals in the agricultural industry in Greece. Moreover, as the use of vaccines would contribute to decreasing the incidence of the various infections and potentially the use of antibiotics in the farms.

In dairy sheep and goats, many of the optional vaccinations scheduled in the farms are carried out during the dry-period, at the end of gestation, because they aim to protect the newborn lambs and kids (e.g., vaccination against clostridial infections, vaccination against pneumonia), as well as the adult animals against mammary infections, given the importance of the mammary glands in those production systems (e.g., vaccination against contagious agalactia and vaccination against staphylococcal mastitis). However, the dry-period in ewes and does is frequently of a short duration, often only up to two-months long; this is a rather short period for accommodating four or five vaccinations with two- to three-week intervals. This, first, creates management problems, as animals are stressed during the last stage of gestation due to the frequent handlings; moreover, it may also raise some concerns regarding the development of efficient immunity after repeated vaccinations. Further work in this area will be valuable and will help the development of effective vaccination schedules in dairy production systems.

## Figures and Tables

**Table 1:** Descriptive findings (number of farms (proportion (95% confidence interval))) regarding administration of vaccines in small ruminant farms in Greece.

Infection against Which Vaccinations Were Made	Frequency of Vaccination		
	Sheep Flocks (*n* = 325)	Goat Herds (*n* = 119)	*p*
Brucellosis ^1^	301 (100% (98.7–100%))	106 (100% (96.5–100%))	-
Chlamydial abortion	130 (40.0% (34.8–45.4%))	39 (32.8% (25.0–41.6%))	0.16
Clostridial infections	316 (97.2% (94.8–98.5%))	118 (99.2% (95.4–99.9%))	0.23
Contagious agalactia	186 (57.2% (51.8–62.5%))	65 (54.6% (45.7–63.3%))	0.62
Contagious ecthyma	3 (0.9% (0.3–2.7%))	1 (0.8% (0.2–4.6%))	0.93
Foot-rot	5 (1.5% (0.7–3.6%))	0 (0.0% (0.0–3.1%))	0.17
Paratuberculosis	11 (3.4% (1.9% 6.0%))	31 (26.1% (19.0–34.6%))	<0.0001
Pneumonia	144 (44.3% (39.0–49.8%))	39 (32.8% (25.0–41.6%))	0.028
Staphylococcal mastitis	126 (38.8% (33.6–44.2%))	34 (28.6% (22.0–37.3%))	0.047
*T. gondii* abortion	0 (0.0% (0.0–1.2%))	0 (0.0% (0.0–3.1%))	n/a ^2^

^1^ compulsory vaccination in the mainland of the country and compulsory non-vaccination in the islands (bar the island of Lesvos, where vaccination is compulsory). ^2^ n/a: not available.

**Table 2:** Most frequent vaccination programs applied in small ruminant farms in Greece.

Infection against Which Vaccinations Were Made	Frequency of Vaccination	
	Sheep Flocks	Goat Herds
Brucellosis	Use in females kept for replacement (*n* = 301, 100% ^1^)	Use in females kept for replacement (*n* = 106, 100%)
Clostridial infections	Use in pregnant females one month before expected start of lambing season (*n* = 275, 91.4%) or one month before expected start of lambing season and six months thereafter (*n* = 12, 4.0%)	Use in pregnant females one month before expected start of kidding season (*n* = 105, 91.3%) or one month before expected start of kidding season and six months thereafter (*n* = 6, 5.2%)
Contagious agalactia	Use in pregnant females three months before expected start of lambing season (*n* = 38, 82.6%) or one week before expected lambing (*n* = 4, 8.7%) or twice during gestation 2–3 months apart (*n* = 4, 8.7%)	Use in pregnant females three months before expected start of kidding season (*n* = 18, 99.0%)
Staphylococcal mastitis	Use in pregnant females during gestation (*n* = 103, 100%)	Use in pregnant females during gestation (*n* = 31, 100%)

^1^ number of farmers who indicated this vaccination schedule to be employed in their farms, proportion among farmers who provided details of vaccination schedules.

**Table 3:** Association of vaccination against contagious agalactia or staphylococcal mastitis with average milk production per animal in small ruminant farms in Greece.

Vaccination Applied	Sheep Flocks	Goat Herds
No vaccination applied	166 ± 7 ^1^ L (*n* = 104)	170 ± 18 L (*n* = 45)
Vaccination applied against contagious agalactia or staphylococcal mastitis	221 ± 8 L (*n* = 130)	216 ± 15 L (*n* = 49)
Vaccination applied against both contagious agalactia and staphylococcal mastitis	236 ± 9 L (*n* = 91)	226 ± 25 L (*n* = 25)
*p*	< 0.0001	0.08

^1^ mean ± standard error of the mean.

**Table 4:** Summary of multivariable analyses for outcomes regarding optional vaccinations in small ruminant farms in Greece.

Outcome	Farm	Variable	*p* Value
Vaccination against chlamydial abortion	S ^1^	Daily number of milking sessions	0.002
		Age of newborns when taken away from dam	0.003
	G ^1^	Average age of culling female animals	0.001
		Period spent daily by farmer at the farm premises	0.002
		Daily number of milking sessions	0.031
		Breed of animals in the farm	0.032
		Age of newborns when taken away from dam	0.037
Vaccination against clostridial infections	S	Management system applied in the farm	0.003
		Family tradition in farming	0.006
		Age of farmers	0.024
	G	Family tradition in farming	0.006
Vaccination against contagious agalactia	S	Management system applied in the farm	0.0002
		Duration of dry period	0.002
		Collaboration with a veterinarian	0.013
	G	Use of laboratory diagnostic examinations in milk samples	0.002
		Collaboration with a veterinarian	0.020
Vaccination against contagious ecthyma	S	Average age of culling female animals	0.041
		Farmer’s general education	0.047
	G	No variables found with a significant association	
Vaccination against foot-rot	S	Farmer’s general education	0.008
Vaccination against paratuberculosis	S	No variables found with a significant association	
	G	No. of female animals in the farm	0.009
Vaccination against pneumonia	S	Routine administration of antibiotics to newborns	0.010
		Period spent daily by farmer at the farm premises	0.017
	G	No variables found with a significant association	
Vaccination against staphylococcal mastitis	S	Use of laboratory diagnostic examinations in milk samples	0.005
		Collaboration with a veterinarian	0.025
Vaccination against staphylococcal mastitis	G	Type of milking mode	0.002
		Daily period spent by farmer at the farm	0.006
		Daily number of milking sessions	0.016

^1^ S: sheep flocks, G: goat herds.

**Table 5:** Detailed results of multivariable analyses for associations with total number of optional vaccinations performed in 325 sheep flocks and 119 goat herds in Greece.

Variables	Regression Coefficients (± Standard Error)
**Sheep flocks**	
Age of farmers (*p* = 0.003)	
per unit change	–0.10 ± 0.01
Management system applied in farms (*p* = 0.005)	
Intensive	0.77 ± 0.09
Semi-intensive	reference
Semi-extensive	–0.77 ± 0.09
Extensive	–1.54 ± 0.18
Daily number of milking sessions (*p* = 0.007)	
per unit change	–1.58 ± 0.17
Age of newborns when taken away from dam (*p* = 0.008)	
per unit change	–0.09 ± 0.01
Use of laboratory diagnostic examinations in milk samples (*p* = 0.015)	
Yes	reference-
No	1.50 ± 0.17
Collaboration with a veterinarian (*p* = 0.025)	
Yes	reference-
No	–0.64 ± 0.20
Routine administration of antibiotics to newborns (*p* = 0.031)	
Yes	reference-
No	–0.70 ± 0.16
**Goat herds**	
Daily period spent by farmer at the farm (*p* = 0.036)	
per unit change	1.07 ± 1.03

**Table 6:** Total number of optional vaccinations performed in 325 sheep flocks and 119 goat herds in Greece in accordance with breeds of animals in these farms.

Type of Breed of Animals in Farms	Sheep Flocks	Goat Herds
Crossbreeds	2.70 ± 0.19 ^1^ (*n* = 43)	3.11 ± 0.29 (*n* = 18)
Imported breeds	3.14 ± 0.10 (*n* = 139)	3.13 ± 0.209 (*n* = 45)
Local breeds	2.59 ± 0.12 (*n* = 143)	2.36 ± 0.18 (*n* = 56)
*p*	0.002	0.008

^1^ mean ± standard error of the mean.

## Data Availability

Most data presented in this study are in the Supplementary Materials. The remaining data are available on request from the corresponding author. The data are not publicly available as they form part of the PhD thesis of the first author, which has not yet been examined, approved, and uploaded in the official depository of PhD theses from Greek Universities.

## Supplementary Materials

The following supporting information can be downloaded at: https://www.mdpi.com/article/10.3390/vaccines10091372/s1, Table S1: Details of variables (*n* = 48) collected during interview of farmers by means of a structured questionnaire and used for the assessment of patterns of vaccinations in 444 small ruminant farms during a countrywide investigation in Greece; Table S2: Details of multivariable models (*n* = 16) employed for the evaluation for potential associations with optional vaccinations in 325 sheep flocks and 119 goat herds in Greece; Table S3: Frequency of usage of specific immunological products for optional vaccinations in 325 sheep flocks and 119 goat herds in Greece; Table S4: Association between the total number of optional vaccinations performed in a farm and the average yearly milk production, as found in 444 small ruminant farms in Greece; Table S5: Results of univariable analysis for associations with optional vaccination against chlamydial abortion in 325 sheep flocks and 119 goat herds in Greece; Table S6: Results of univariable analysis for associations with optional vaccination against clostridial infections in 325 sheep flocks and 119 goat herds in Greece; Table S7: Results of univariable analysis for associations with optional vaccination against contagious agalactia in 325 sheep flocks and 119 goat herds in Greece; Table S8: Results of univariable analysis for associations with optional vaccination against contagious ecthyma in 325 sheep flocks and 119 goat herds in Greece, Table S9: Results of univariable analysis for associations with optional vaccination against foot-rot in 325 sheep flocks in Greece; Table S10: Results of univariable analysis for associations with optional vaccination against paratuberculosis in 325 sheep flocks and 119 goat herds in Greece; Table S11: Results of univariable analysis for associations with optional vaccination against pneumonia in 325 sheep flocks and 119 goat herds in Greece; Table S12: Results of univariable analysis for associations with optional vaccination against staphylococcal mastitis in 325 sheep flocks and 119 goat herds in Greece; Table S13: Results of univariable analysis for associations with total number of optional vaccinations in 325 sheep flocks and 119 goat herds in Greece.

## Appendix A

**Table A1:** Multivariable analysis for variables associated with optional vaccination against chlamydial abortion in 444 small ruminant farms in Greece.

Variables	Odds Ratio ^1^ (95% Confidence Intervals)	*p* Value
(a) Sheep flocks		
Daily number of milking sessions		0.002
One (0/1 = 0.0%)	5.133 (0.201–131.421)	0.32
Two (92/ 264 = 34.8%)	3.229 (1.803–5.785)	0.0001
Three (38/60 = 63.3%)	reference	-
Age of newborns when taken away from dam		0.003
≤40 days (63/117 = 53.8%)	reference	-
41–60 days (61/172 = 35.5%)	2.123 (1.315–3.428)	0.002
>6.5 days (6/36 = 16.7%)	5.833 (2.258–15.067)	0.0003
(b) Goat herds		
Average age of culling female animals		0.001
≤6.5 years (26/50 = 52.0%)	reference	-
>6.5 years (13/69 = 18.8%)	4.667 (2.056–10.593)	0.0002
Daily period spent by farmer at the farm		0.002
≤8 h (5/28 = 17.9%)	2.744 (0.954–7.890)	0.06
>8 h (34/91 = 37.4%)	reference	-
Daily number of milking sessions		0.031
One (1/4 = 25.0%)	18.000 (0.812–399.177)	0.07
Two (32/108 = 29.6%)	14.500 (1.648–123.188)	0.016
Three (6/7 = 85.7%)	reference	-
Breed of animals in the farm		0.032
Crossbreeds (7/18 = 38.9%)	0.971 (0.326–2.895)	0.957
Imported breeds (21/45 = 46.7%)	reference	-
Local breeds (11/55 = 20.0%)	2.471 (1.050–5.814)	0.038
Age of newborns when taken away from dam		0.037
≤40 days (15/26 = 57.7%)	reference	-
41–60 days (12/44 = 27.3%)	3.636 (1.308–10.110)	0.013
>60 days (12/49 = 24.5%)	4.205 (1.524–11.597)	0.006

^1^ Odds ratios calculated against the lowest prevalence associations of the variable.

**Table A2:** Multivariable analysis for variables associated with optional vaccination against clostridial infections in 444 small ruminant farms in Greece.

Variables	Odds Ratio ^1^ (95% Confidence Intervals)	*p* Value
(a) Sheep flocks		
Management system applied in the farm		0.003
Intensive (44/44 = 100.0%)	reference	-
Semi-intensive (138/140 = 95.0%)	1.607 (0.076–34.097)	0.76
Semi-extensive (112/116 = 96.6%)	3.560 (0.188–67.498)	0.40
Extensive (22/25 = 88.0%)	13.844 (0.685–279.832)	0.09
Family tradition in farming		0.006
Yes (277/283 = 97.9%)	reference	-
No (39/42 = 92.9%)	4.735 (0.767–29.234)	0.09
Age of farmers		0.024
≤50 years (195/197 = 99.0%)	reference	-
>50 years (121/128 = 94.5%)	0.460 (0.094–2.253)	0.34
(b) Goat herds		
Family tradition in farming		0.006
Yes (103/103 = 100.0%)	reference	-
No (15/16 = 93.8%)	20.032 (0.781–513.999)	0.07

^1^ Odds ratios calculated against the lowest prevalence associations of the variable.

**Table A3:** Multivariable analysis for variables associated with optional vaccination against contagious agalactia in 444 small ruminant farms in Greece.

Variables	Odds Ratio ^1^ (95% Confidence Intervals)	*p* Value
(a) Sheep flocks		
Management system applied in the farm		0.0004
Intensive (36/44 = 81.8%)	reference	-
Semi-intensive (89/140 = 63.6%)	2.579 (1.113–5.972)	0.027
Semi-extensive (58/116 = 50.0%)	4.500 (1.927–10.509)	0.0005
Extensive (3/25 = 12.0%)	33.000 (7.905–137.760)	<0.0001
Duration of dry period		0.013
≤2 months (77/107 = 72.0%)	reference	-
>2 months (109/218 = 50.0%)	2.567 (1.559–4.225)	0.0002
Collaboration with a veterinarian		0.032
Yes (173/283 = 61.1%)	reference	-
No (13/42 = 31.0%)	3.508 (1.748–7.040)	0.0004
(b) Goat herds		
Use of laboratory diagnostic examinations in samples of milk		0.009
Yes (22/25 = 88.0%)	reference	-
No (43/94 = 45.7%)	8.698 (2.436–31.056)	0.0009
Collaboration with a veterinarian		0.034
Yes (62/102 = 60.8%)	reference	-
No (3/17 = 17.6%)	7.233 (1.954–26.776)	0.003

^1^ Odds ratios calculated against the lowest prevalence associations of the variable.

**Table A4:** Multivariable analysis for variables associated with optional vaccination against contagious ecthyma in 444 small ruminant farms in Greece.

Variables	Odds Ratio ^1^ (95% Confidence Intervals)	*p* Value
(a) Sheep flocks		
Average age of culling female animals		0.041
≤6.5 years (1/242 = 0.4%)	5.951 (0.533–66.497)	0.15
>6.5 years (2/83 = 2.4%)	reference	-
Farmer’s general education		0.047
Primary education (2/57 = 3.5%)	reference	-
Secondary or post-secondary education (1/225 = 0.4%)	8.146 (0.725–91.470)	0.09
Tertiary education (0/43 = 0.0%)	3.919 (0.183–83.769)	0.38
(b) Goat herds		
No variables found with a significant association		

^1^ Odds ratios calculated against the lowest prevalence associations of the variable.

**Table A5:** Multivariable analysis for variables associated with optional vaccination against foot-rot in 325 sheep farms in Greece.

Variable	Odds Ratio ^1^ (95% Confidence Intervals)	*p* Value
Farmer’s general education		0.008
Primary education (0/57 = 0.0%)	9.938 (0.500–197.708)	0.13
Secondary or post-secondary education (2/225 = 0.9%)	8.363 (1.354–51.643)	0.022
Tertiary education (3/43 = 7.0%)	reference	-

^1^ Odds ratios calculated against the lowest prevalence associations of the variable.

**Table A6:** Multivariable analysis for variables associated with optional vaccination against paratuberculosis in 444 small ruminant farms in Greece.

Variables	Odds Ratio ^1^ (95% Confidence Intervals)	*p* Value
(a) Sheep flocks		
No variables found with a significant association		
(b) Goat herds		
No. of female animals in the farm		0.0009
≤165 does (8/54 = 14.8%)	6.708 (1.785–25.211)	0.005
166–330 does (11/37 = 29.7%)	2.758 (0.753–10.103)	0.13
331–500 does (5/13 = 38.5%)	1.867 (0.392–8.895)	0.43
>500 does (7/13 = 53.8%)	reference	-

^1^ Odds ratios calculated against the lowest prevalence associations of the variable.

**Table A7:** Multivariable analysis for variables associated with optional vaccination against pneumonia in 444 small ruminant farms in Greece.

Variables	Odds Ratio ^1^ (95% Confidence Intervals)	*p* Value
(a) Sheep flocks		
Routine administration of antibiotics to newborns		0.010
Yes (41/70 = 58.6%)	reference	-
No (103/255 = 40.4%)	2.086 (1.219–3.571)	0.007
Daily period spent by farmer at the farm		0.017
≤8 h (34/99 = 34.3%)	1.813 (1.111–2.959)	0.017
>8 h (110/226 = 48.7%)	reference	-
(b) Goat herds		
No variables found with a significant association		

^1^ Odds ratios calculated against the lowest prevalence associations of the variable.

**Table A8:** Multivariable analysis for variables associated with optional vaccination against staphylococcal mastitis in 444 small ruminant farms in Greece.

Variables	Odds Ratio ^1^ (95% Confidence Intervals)	*p* Value
(a) Sheep flocks		
Use of laboratory diagnostic examinations in samples of milk		0.005
Yes (39/70 = 55.7%)	reference	-
No (87/255 = 34.1%)	2.429 (1.419–4.161)	0.001
Collaboration with a veterinarian		0.025
Yes (119/283 = 42.0%)	reference	-
No (7/42 = 16.7%)	3.628 (1.558–8.447)	0.003
(b) Goat herds		
Type of milking mode		0.002
Machine-milking (27/66 = 40.9%)	reference	-
Hand-milking (7/53 = 13.2%)	4.550 (1.787–11.581)	0.002
Daily period spent by farmer at the farm		0.006
≤8 h (5/28 = 17.9%)	2.152 (0.743–6.228)	0.16
>8 h (29/91 = 31.9%)	reference	-
Daily number of milking sessions		0.016
One (0/4 = 0.0%)	19.800 (0.744–527.292)	0.07
Two (29/108 = 26.9%)	6.810 (1.251–37.063)	0.027
Three (5/7 = 71.4%)	reference	-

^1^ Odds ratios calculated against the lowest prevalence associations of the variable.
