# Supplementary material for: Administration of Vaccines in Dairy Sheep and Goat Farms: Patterns of Vaccination, Associations with Health and Production Parameters, Predictors
Source: Vaccines (Basel). 2022 Aug 23;10(9):1372. doi: 10.3390/vaccines10091372 (PMC9502793; doi:10.3390/vaccines10091372)
Supplement: Supplementary file 1 [file vaccines-10-01372-s001.zip › vaccines-1842575-SI.pdf]

Administration of Vaccines in Dairy Sheep and Goat Farms: Patterns of Vaccination, Associations with Health and Production Parameters, Predictors

Daphne T. Lianou, Charalambia K. Michael, Efthymia Petinaki, Vasia S. Mavrogianni and George C. Fthenakis

Table S1. Details of variables (n = 48) collected during interview of farmers by means of a structured questionnaire and used for the assessment of patterns of vaccinations in 444 small ruminant farms during a countrywide investigation in Greece.

Management system applied in the farm (description according to EFSA classification [1])
Total surface of land available to animals for grazing (acres)
Material of the floor of the barn (soil / other)
Availability of straw bedding (yes / no)
Type of milking mode (hand-milking / machine-milking)
No. of female animals in the farm (no.)
Breed of animals in the farm (description)
Average age of culling female animals (years)
Month of the start of the lambing / kidding season (description)
Total milk quantity per ewe / doe obtained during the preceding milking period (litres)
Total number of lambs / kids born during the preceding lambing / kidding season (no.)
Total number of lambs / kids sold during the preceding lambing / kidding season (no.)
Total number of adult animals that died during the preceding production year (no.)
Collaboration with a veterinarian (yes / no)
Age of newborns when taken away from dam (days)
Application of teat disinfection after milking (yes / no)
Use of laboratory diagnostic examinations in samples of milk (yes / no)
Administration of 'dry-ewe' treatment at the end of the lactation period (yes / no)
Duration of dry-period (months)
Daily number of milking sessions (no.)
Vaccination against brucellosis (yes / no)
Immunological product used for vaccination against brucellosis and vaccination regime applied (description)
Vaccination against chlamydial abortion (yes / no)
Immunological product used for vaccination against chlamydial abortion and vaccination regime applied (description)
Vaccination against clostridial infections (yes / no)
Immunological product used for vaccination against clostridial infections and vaccination regime applied (description)
Vaccination against contagious agalactia (yes / no)
Immunological product used for vaccination against contagious agalactia and vaccination regime applied (description)
Vaccination against contagious ecthyma (yes / no)

Immunological product used for vaccination against contagious ecthyma and vaccination regime applied (description)
Vaccination against foot-rot (yes / no)
Immunological product used for vaccination against foot-rot and vaccination regime applied (description)
Vaccination against paratuberculosis (yes / no)
Immunological product used for vaccination against paratuberculosis and vaccination regime applied (description)
Vaccination against pneumonia (yes / no)
Immunological product used for vaccination against pneumonia and vaccination regime applied (description)
Vaccination against staphylococcal mastitis (yes / no)
Immunological product used for vaccination against staphylococcal mastitis and vaccination regime applied (description)
Vaccination against *Toxoplasma gondii* abortion (yes / no)
Immunological product used for vaccination against *T. gondii* abortion and vaccination regime applied (description)
Routine administration of antibiotics to newborns (yes / no)
Age of farmer (years)
Length of previous animal farming experience (years)
Farmer's general education (description)
Farmer's professional involvement in farming (full-time / part-time)
Daily period spent by farmer at the farm (hours)
Family tradition in farming (yes / no)
Presence of working staff in the farm (yes/no)

[1] management system classified as intensive, semi-intensive, semi-extensive, extensive (European Food Safety Authority. Scientific opinion on the welfare risks related to the farming of sheep for wool, meat and milk production. *EFSA J.* **2014**, *12*, 3933–4060.).

Table S2. Details of multivariable models (*n* = 16) employed for the evaluation for potential associations with optional vaccinations in 325 sheep flocks and 119 goat herds in Greece.

Outcome	Variables (*n*)		
	**assessed in univariable analyses**	**offered to the multivariable models**	**required in the final models**
Vaccination against chlamydial abortion - sheep	16	9	(a) Age of newborns when taken away from dam, (b) Daily number of milking sessions, (c) Age of farmers, (d) Presence of working staff in the farm
Vaccination against chlamydial abortion - goats	16	7	(a) Breed of animals in the farm, (b) Average age of culling female animals, (c) Age of newborns when taken away from dam, (d) Daily number of milking sessions, (e) Daily period spent by farmer at the farm
Vaccination against clostridial infections – sheep	19	6	(a) Management system applied in the farm, (b) Age of farmers, (c) Family tradition in farming
Vaccination against clostridial infections - goats	19	4	(a) Routine administration of antibiotics to newborns, (b) Family tradition in farming
Vaccination against contagious agalactia -sheep	21	16	(a) Management system applied in the farm, (b) Collaboration with a veterinarian, (c) Age of newborns when taken away from dam, (d) Use of laboratory diagnostic examinations in samples of milk, (e) Administration of 'dry -ewe' treatment at the end of the lactation period, (f) Duration of dry-period
Vaccination against contagious agalactia - goats	21	17	(a) Type of milking mode, (b) Breed of animals in the farm, (c) Collaboration with a veterinarian, (d) Use of laboratory diagnostic examinations in samples of milk, (e) Administration of 'dry -ewe' treatment at the end of the lactation period, (f) Daily number of milking sessions, (g) Farmer's general education, (h) Farmer's professional involvement in farming

Vaccination against contagious ecthyma - sheep	15	6	(a) No. of female animals in the farm, (b) Average age of culling female animals, (c) Farmer's general education, (d) Daily period spent by farmer at the farm, (e) Presence of working staff in the farm
Vaccination against contagious ecthyma - goats	15	4	(a) Farmer's general education, (b) Daily period spent by farmer at the farm, (c) Presence of working staff in the farm
Vaccination against footrot - sheep	19	3	(a) Farmer's professional involvement in farming, (b) Daily period spent by farmer at the farm
Vaccination against paratuberculosis - sheep	19	6	(a) Type of milking mode, (b) Average age of culling female animals, (c) Month of the start of the lambing season
Vaccination against paratuberculosis - goats	19	6	(a) Material of the floor of the barn, (b) No. of female animals in the farm
Vaccination against pneumonia - sheep	18	5	(a) Total surface of land available to animals for grazing, (b) Month of the start of the lambing season, (c) Routine administration of antibiotics to newborns, (d) Daily period spent by farmer at the farm
Vaccination against pneumonia - goats	18	2	(a) Presence of working staff in the farm
Vaccination against staphylococcal mastitis - sheep	21	14	(a) Management system applied in the farm, (b) Collaboration with a veterinarian, (c) Use of laboratory diagnostic examinations in samples of milk, (d) Administration of 'dry-ewe' treatment at the end of the lactation period, (e) Farmer's professional involvement in farming
Vaccination against staphylococcal mastitis - goats	21	11	(a) Type of milking mode, (b) Age of newborns when taken away from dam, (c) Daily number of milking sessions, (d) Daily period spent by farmer at the farm

Total number of optional vaccines administered - sheep	19	14	(a) Management system applied in the farm, (b) Collaboration with a veterinarian, (c) Age of newborns when taken away from dam, (d) Routine administration of antibiotics to newborns, (e) Daily number of milking sessions, (f) Use of laboratory diagnostic examinations in samples of milk, (g) Administration of 'dry-ewe' treatment at the end of the lactation period, (h) Age of farmers
Total number of optional vaccines administered - goat	16	10	(a) Type of milking mode, (b) Breed of animals in the farm, (c) Daily number of milking se-ssions, (d) Use of laboratory diagnostic examinations in samples of milk, (e) Administration of 'dry -ewe' treatment at the end of the lactation period, (f) Daily period spent by farmer at the farm

Table S3. Frequency of usage of specific immunological products for optional vaccinations in 325 sheep flocks and 119 goat herds in Greece.

**Vaccinations against chlamydial abortion**		
	Farmers who provided the information	
Vaccine used [3]	Sheep flocks (n = 101) [1]	Goat herds (n = 31)
Enzovax	98 (97.0%) [2]	29 (93.5%)
Inmeva	1 (1.0%)	1 (3.2%)
Ovax	2 (2.0%)	1 (3.2%)
**Vaccinations against clostridial infections**		
	Farmers who provided the information	
Vaccine used [3]	Sheep flocks (n = 295)	Goat herds (n = 108)
Anero	6 (2.0%)	9 (8.3%)
Bravoxin	64 (21.7%)	32 (29.6%)
Covexin 8A	90 (30.5%)	37 (34.3%)
Covexin 10	1 (0.3%)	0 (0.0%)
Cubolac	3 (1.0%)	0 (0.0%)
Dialuene P	126 (42.7%)	32 (29.6%)
Panclostil	16 (5.4%)	9 (8.3%)
Toxipra	67 (22.7%)	29 (26.9%)
**Vaccinations against contagious agalactia**		
	Farmers who provided the information	
Vaccine used [3]	Sheep flocks (n = 144)	Goat herds (n = 52)
Agalax	120 (83.3%)	39 (75.0%)
Galazel	32 (22.2%)	14 (26.9%)
**Vaccinations against contagious ecthyma**		
	Farmers who provided the information	
	Sheep flocks (n = 0)	Goat herds (n = 0)
**Vaccinations against foot-rot**		
	Farmers who provided the information	
Vaccine used [3]	Sheep flocks (n = 4)	Goat herds (n = 0)
Footvax	4 (100.0%)	
**Vaccinations against paratuberculosis**		
	Farmers who provided the information	
Vaccine used [3]	Sheep flocks (n = 3)	Goat herds (n = 26)
Gudair	3 (100.0%)	26 (100.0%)
**Vaccinations against pneumonia**		
	Farmers who provided the information	
Vaccine used [3]	Sheep flocks (n = 139)	Goat herds (n = 39)
Dialuene P	126 (90.6%)	33 (84.6%)
Ovipast	16 (11.5%)	8 (20.5%)

**Vaccinations against staphylococcal mastitis**		
	Farmers who provided the information	
Vaccine used [3]	Sheep flocks (*n* = 124)	Goat herds (*n* = 34)
Mastivet	14 (11.3%)	3 (8.8%)
Ovax	21 (16.9%)	6 (17.6%)
Startvac	9 (7.3%)	1 (2.9%)
Vimco	81 (65.3%)	25 (73.5%)
**Vaccinations against *T. gondii* abortion**		
	Farmers who provided the information	
	Sheep flocks (*n* = 0)	Goat herds (*n* = 0)

[1] number of sheep flocks or goat herds in which specific products used for vaccinations were reported.
[2] number of farms (proportion).
[3] all product names listed are ®.

Table S4. Association between the total number of optional vaccinations performed in a farm and the average yearly milk production, as found in 444 small ruminant farms in Greece.

	Number of vaccines administered						
	0 (*n* = 4 [1])	1 (*n* = 18)	2 (*n* = 57)	3 (*n* = 121)	4 (*n* = 111)	5 (*n* = 77)	6 (*n* = 47)
Average milk production (L)	62.0 ± 5.5	112.9 ± 15.0	181.7 ± 10.4	190.0 ± 9.4	221.3 ± 9.0	219.8 ± 8.7	256.8 ± 13.6

[1] number of farms, in which respective number of vaccinations were performed.

Table S5. Results of univariable analysis for associations with optional vaccination against chlamydial abortion in 325 sheep flocks and 119 goat herds in Greece.

(a) Sheep flocks

**Vaccination performed (*n* = 130)**				**Vaccination not performed (*n* = 195)**				***p***
**Management system applied in the farm**								
Intensive	Semi-intensive	Semi-extensive	Extensive	Intensive	Semi-intensive	Semi-extensive	Extensive	
24	66	35	5	20	74	81	20	0.001
**No. of ewes in the flock**								
≤ 165 ewes	166-330 ewes	331-500 ewes	> 500 ewes	≤ 165 ewes	166-330 ewes	331-500 ewes	> 500 ewes	
28	46	30	26	60	74	36	25	0.11
**Breed of animals**								
Crossbreeds	Imported breeds	Local breeds		Crossbreeds	Imported breeds	Local breeds		
17	61	52		26	78	91		0.43
**Milking mode in flock**								
Machine-milking	Hand-milking			Machine-milking	Hand-milking			
118	12			137	58			< 0.0001
**Average age of culling ewes**								
≤ 6.5 years	> 6.5 years			≤ 6.5 years	> 6.5 years			
104	26			138	57			0.06
**Start of lambing period**								
All year	Autumn	Winter	Spring - Summer	All year	Autumn	Winter	Spring - Summer	
7	106	15	2	11	139	40	5	0.16
**Collaboration with a veterinarian**								
Yes	No			Yes	No			
117	13			166	29			0.20
**Age of newborns taken away from dam**								
≤ 40 days	41 – 60 days	≥ 61 days		≤ 40 days	41 – 60 days	≥ 61 days		
63	61	6		54	111	30		< 0.0001
**Number of daily milking sessions**								
One	Two	Three		One	Two	Three		
0	92	38		1	172	22		0.0002
**Age of the farmer**								
Up to 50 years	Over 50 years			Up to 50 years	Over 50 years			
91	39			106	89			0.005

**Length of previous animal farming experience of the farmer**						
≤ 5 years	> 5 years		≤ 5 years	> 5 years		
34	96		40	155		0.23
**Education of the farmer**						
Primary education	Secondary or post-secondary education	Tertiary education	Primary education	Secondary or post-secondary education	Tertiary education	
25	87	18	32	138	25	0.75
**Professional involvement in farming**						
Full-time	Part-time		Full-time	Part-time		
116	14		176	19		0.76
**Daily period at the farm**						
≤ 8 hours	> 8 hours		≤ 8 hours	> 8 hours		
36	94		63	132		0.38
**Family tradition in farming**						
Yes	No		Yes	No		
112	18		171	24		0.69
**Presence of working staff in the flock**						
Yes	No		Yes	No		
63	67		60	135		0.002

(b) Goat herds

**Vaccination performed (*n* = 39)**				**Vaccination not performed (*n* = 80)**				***p***
**Management system applied in the farm**								
Intensive	Semi-intensive	Semi-extensive	Extensive	Intensive	Semi-intensive	Semi-extensive	Extensive	
2	12	21	4	7	17	40	16	0.39
**No. of does in the herd**								
≤ 165 does	166-330 does	331-500 does	> 500 does	≤ 165 does	166-330 does	331-500 does	> 500 does	
18	11	7	3	38	26	6	10	0.34
**Breed of animals**								
Crossbreeds	Imported breeds	Local breeds		Crossbreeds	Imported breeds	Local breeds		
7	21	11		11	24	45		0.013
**Milking mode in herd**								
Machine-milking	Hand-milking			Machine-milking	Hand-milking			
26	13			40	40			0.09

**Average age of culling does**								
≤ 6.5 years	> 6.5 years	≤ 6.5 years	> 6.5 years					
26	13	24	56					0.0001
**Start of kidding period**								
All year	Autumn	Winter	Spring - Summer	All year	Autumn	Winter	Spring - Summer	
3	23	12	1	5	40	32	3	0.75
**Collaboration with a veterinarian**								
Yes	No	Yes	No					
36	3	65	15					0.11
**Age of newborns taken away from dam**								
≤ 40 days	41 – 60 days	≥ 61 days	≤ 40 days	41 – 60 days	≥ 61 days			
15	12	12	11	32	37			0.009
**Number of daily milking sessions**								
One	Two	Three	One	Two	Three			
1	32	6	3	76	1			0.009
**Age of the farmer**								
Up to 50 years	Over 50 years	Up to 50 years	Over 50 years					
26	13	47	33					0.41
**Length of previous animal farming experience of the farmer**								
≤ 5 years	> 5 years	≤ 5 years	> 5 years					
9	30	15	65					0.58
**Education of the farmer**								
Primary education	Secondary or post-secondary education	Tertiary education	Primary education	Secondary or post-secondary education	Tertiary education			
4	30	5	16	59	5			0.24
**Professional involvement in farming**								
Full-time	Part-time	Full-time	Part-time					
36	3	69	11					0.34
**Daily period at the farm**								
≤ 8 hours	> 8 hours	≤ 8 hours	> 8 hours					
5	34	23	57					0.05
**Family tradition in farming**								
Yes	No	Yes	No					
33	6	70	10					0.67

Presence of working staff in the flock				
Yes	No	Yes	No	
11	28	23	57	0.95

Table S6. Results of univariable analysis for associations with optional vaccination against clostridial infections in 325 sheep flocks and 119 goat herds in Greece.

(a) Sheep flocks

**Vaccination performed (*n* = 316)**				**Vaccination not performed (*n* = 9)**				***p***
**Management system applied in the farm**								
Intensive	Semi-intensive	Semi-extensive	Extensive	Intensive	Semi-intensive	Semi-extensive	Extensive	
44	138	112	22	0	2	4	3	0.016
**No. of ewes in the flock**								
≤ 165 ewes	166-330 ewes	331-500 ewes	> 500 ewes	≤ 165 ewes	166-330 ewes	331-500 ewes	> 500 ewes	
86	116	64	50	2	4	2	1	0.95
**Breed of animals**								
Crossbreeds	Imported breeds	Local breeds		Crossbreeds	Imported breeds	Local breeds		
41	138	137		2	1	6		0.15
**Total land available for grazing (acres per animal)**								
≤ 0.50	0.51 – 2.00	≥ 2.00		≤ 0.50	0.51 – 2.00	≥ 2.00		
116	126	74		2	4	3		0.63
**Use of straw as bedding material**								
Yes	No			Yes	No			
262	54			6	3			0.21
**Milking mode in flock**								
Machine-milking	Hand-milking			Machine-milking	Hand-milking			
249	67			6	3			0.38
**Average age of culling ewes**								
≤ 6.5 years	> 6.5 years			≤ 6.5 years	> 6.5 years			
235	81			7	2			0.82
**Start of lambing period**								
All year	Autumn	Winter	Spring - Summer	All year	Autumn	Winter	Spring - Summer	
18	237	54	7	0	8	1	0	0.77
**Collaboration with a veterinarian**								
Yes	No			Yes	No			
277	39			6	3			0.06
**Age of newborns taken away from dam**								
≤ 40 days	41 – 60 days	≥ 61 days		≤ 40 days	41 – 60 days	≥ 61 days		
114	170	32		3	2	4		0.004

**Routine administration of antibiotics to newborns**						
Yes	No		Yes	No		
68	248		2	7		0.96
**Number of daily milking sessions**						
One	Two	Three	One	Two	Three	
1	255	60	0	9	0	0.34
**Age of the farmer**						
Up to 50 years	Over 50 years		Up to 50 years	Over 50 years		
195	121		2	7		0.017
**Length of previous animal farming experience of the farmer**						
≤ 5 years	> 5 years		≤ 5 years	> 5 years		
73	243		1	8		0.40
**Education of the farmer**						
Primary education	Secondary or post-secondary education	Tertiary education	Primary education	Secondary or post-secondary education	Tertiary education	
57	217	42	0	8	1	0.34
**Professional involvement in farming**						
Full-time	Part-time		Full-time	Part-time		
284	32		8	1		0.92
**Daily period at the farm**						
≤ 8 hours	> 8 hours		≤ 8 hours	> 8 hours		
95	221		4	5		0.36
**Family tradition in farming**						
Yes	No		Yes	No		
277	39		6	3		0.06
**Presence of working staff in the flock**						
Yes	No		Yes	No		
120	196		3	6		0.78

(b) Goat herds

Vaccination performed (*n* = 118)				Vaccination not performed (*n* = 1)				*p*
**Management system applied in the farm**								
Intensive	Semi-intensive	Semi-extensive	Extensive	Intensive	Semi-intensive	Semi-extensive	Extensive	
9	29	60	20	0	0	1	0	0.81
**No. of does in the herd**								
≤ 165 does	166-330 does	331-500 does	> 500 does	≤ 165 does	166-330 does	331-500 does	> 500 does	
56	36	13	13	0	1	0	0	0.53
**Breed of animals**								
Crossbreeds	Imported breeds	Local breeds		Crossbreeds	Imported breeds	Local breeds		
18	45	55		0	0	1		0.57
**Total land available for grazing (acres per animal)**								
≤ 0.50	0.51 – 2.00	≥ 2.00		≤ 0.50	0.51 – 2.00	≥ 2.00		
17	31	70		0	0	1		0.37
**Use of straw as bedding material**								
Yes	No			Yes	No			
75	43			1	0			0.45
**Milking mode in herd**								
Machine-milking	Hand-milking			Machine-milking	Hand-milking			
65	53			1	0			0.37
**Average age of culling does**								
≤ 6.5 years	> 6.5 years			≤ 6.5 years	> 6.5 years			
49	69			1	0			0.24
**Start of kidding period**								
All year	Autumn	Winter	Spring - Summer	All year	Autumn	Winter	Spring - Summer	
8	62	44	4	0	1	0	0	0.83
**Collaboration with a veterinarian**								
Yes	No			Yes	No			
100	18			1	0			0.67
**Age of newborns taken away from dam**								
≤ 40 days	41 – 60 days	≥ 61 days		≤ 40 days	41 – 60 days	≥ 61 days		
25	44	49		1	0	0		0.16
**Routine administration of antibiotics to newborns**								
Yes	No			Yes	No			
29	89			1	0			0.08

**Number of daily milking sessions**						
One	Two	Three	One	Two	Three	
4	107	7	0	1	0	0.95
**Age of the farmer**						
Up to 50 years	Over 50 years		Up to 50 years	Over 50 years		
72	46		1	0		0.43
**Length of previous animal farming experience of the farmer**						
≤ 5 years	> 5 years		≤ 5 years	> 5 years		
24	94		0	1		0.61
**Education of the farmer**						
Primary education	Secondary or post-secondary education	Tertiary education	Primary education	Secondary or post-secondary education	Tertiary education	
20	88	10	0	1	0	0.84
**Professional involvement in farming**						
Full-time	Part-time		Full-time	Part-time		
104	14		1	0		0.71
**Daily period at the farm**						
≤ 8 hours	> 8 hours		≤ 8 hours	> 8 hours		
27	91		1	0		0.07
**Family tradition in farming**						
Yes	No		Yes	No		
103	15		0	1		0.011
**Presence of working staff in the flock**						
Yes	No		Yes	No		
34	84		0	1		0.53

Table S7. Results of univariable analysis for associations with optional vaccination against contagious agalactia in 325 sheep flocks and 119 goat herds in Greece.

(a) Sheep flocks

**Vaccination performed (*n* = 186)**				**Vaccination not performed (*n* = 139)**				***p***
**Management system applied in the farm**								
Intensive	Semi-intensive	Semi-extensive	Extensive	Intensive	Semi-intensive	Semi-extensive	Extensive	
36	89	58	3	8	51	58	22	< 0.0001
**No. of ewes in the flock**								
≤ 165 ewes	166-330 ewes	331-500 ewes	> 500 ewes	≤ 165 ewes	166-330 ewes	331-500 ewes	> 500 ewes	
45	72	38	31	43	48	28	20	0.58
**Breed of animals**								
Crossbreeds	Imported breeds	Local breeds		Crossbreeds	Imported breeds	Local breeds		
23	94	69		20	44	75		0.002
**Total land available for grazing (acres per animal)**								
≤ 0.50	0.51 – 2.00	≥ 2.00		≤ 0.50	0.51 – 2.00	≥ 2.00		
80	69	37		38	61	40		0.011
**Milking mode in flock**								
Machine-milking	Hand-milking			Machine-milking	Hand-milking			
163	23			92	47			< 0.0001
**Average age of culling ewes**								
≤ 6.5 years	> 6.5 years			≤ 6.5 years	> 6.5 years			
146	40			96	43			0.05
**Start of lambing period**								
All year	Autumn	Winter	Spring – Summer	All year	Autumn	Winter	Spring - Summer	
11	141	30	4	7	104	25	3	0.96
**Collaboration with a veterinarian**								
Yes	No			Yes	No			
173	13			110	29			0.0002
**Age of newborns taken away from dam**								
≤ 40 days	41 – 60 days	≥ 61 days		≤ 40 days	41 – 60 days	≥ 61 days		
79	97	10		38	75	26		0.0001
**Number of daily milking sessions**								
One	Two	Three		One	Two	Three		
0	145	41		1	119	19		0.08

**Use of laboratory diagnostic examinations in samples of milk**				
Yes	No	Yes	No	
52	134	18	121	0.001
**Application of teat disinfection during milking**				
Yes	No	Yes	No	
33	153	19	120	0.32
**Application of 'dry-period treatment'**				
Yes	No	Yes	No	
40	146	13	126	0.003
**Duration of dry-period (months)**				
≤ 2	> 2	≤ 2	> 2	
77	109	30	109	0.0002
**Age of the farmer**				
Up to 50 years	Over 50 years	Up to 50 years	Over 50 years	
132	54	65	74	< 0.0001
**Length of previous animal farming experience of the farmer**				
≤ 5 years	> 5 years	≤ 5 years	> 5 years	
52	134	22	117	0.01

**Education of the farmer**						
Primary education	Secondary or post-secondary education	Tertiary education	Primary education	Secondary or post-secondary education	Tertiary education	
34	129	23	23	96	20	0.83

**Professional involvement in farming**				
Full-time	Part-time	Full-time	Part-time	
164	22	128	11	0.25
**Daily period at the farm**				
≤ 8 hours	> 8 hours	≤ 8 hours	> 8 hours	
62	124	37	102	0.19
**Family tradition in farming**				
Yes	No	Yes	No	
156	30	127	12	0.046
**Presence of working staff in the flock**				
Yes	No	Yes	No	
83	103	41	98	0.005

(b) Goat herds

Vaccination performed (*n* = 65)				Vaccination not performed (*n* = 54)				*p*
**Management system applied in the farm**								
Intensive	Semi-intensive	Semi-extensive	Extensive	Intensive	Semi-intensive	Semi-extensive	Extensive	
7	17	33	8	2	12	28	12	0.28
**No. of does in the herd**								
≤ 165 ewes	166-330 ewes	331-500 ewes	> 500 ewes	≤ 165 ewes	166-330 ewes	331-500 ewes	> 500 ewes	
32	20	7	6	24	17	6	7	0.91
**Breed of animals**								
Crossbreeds	Imported breeds	Local breeds		Crossbreeds	Imported breeds	Local breeds		
14	29	22		4	16	34		0.004
**Total land available for grazing (acres per animal)**								
≤ 0.50	0.51 – 2.00	≥ 2.00		≤ 0.50	0.51 – 2.00	≥ 2.00		
12	14	39		5	17	32		0.24
**Milking mode in herd**								
Machine-milking	Hand-milking			Machine-milking	Hand-milking			
44	21			22	32			0.003
**Average age of culling does**								
≤ 6.5 years	> 6.5 years			≤ 6.5 years	> 6.5 years			
33	32			17	37			0.034
**Start of kidding period**								
All year	Autumn	Winter	Spring - Summer	All year	Autumn	Winter	Spring - Summer	
7	31	24	3	1	32	20	1	0.18
**Collaboration with a veterinarian**								
Yes	No			Yes	No			
62	3			40	14			0.0009
**Age of newborns taken away from dam**								
≤ 40 days	41 – 60 days	≥ 61 days		≤ 40 days	41 – 60 days	≥ 61 days		
19	22	24		7	22	25		0.10
**Number of daily milking sessions**								
One	Two	Three		One	Two	Three		
1	58	6		3	50	1		0.12
**Use of laboratory diagnostic examinations in samples of milk**								
Yes	No			Yes	No			
22	43			3	51			0.0002

**Application of teat disinfection during milking**						
Yes	No		Yes	No		
11	54		1	53		0.007
**Application of 'dry-period treatment'**						
Yes	No		Yes	No		
14	51		1	53		0.001
**Duration of dry-period (months)**						
≤ 2	> 2		≤ 2	> 2		
20	45		19	35		0.61
**Age of the farmer**						
Up to 50 years	Over 50 years		Up to 50 years	Over 50 years		
44	21		29	25		0.12
**Length of previous animal farming experience of the farmer**						
≤ 5 years	> 5 years		≤ 5 years	> 5 years		
18	47		6	48		0.025
**Education of the farmer**						
Primary education	Secondary or post-secondary education	Tertiary education	Primary education	Secondary or post-secondary education	Tertiary education	
10	51	4	10	35	9	0.14
**Professional involvement in farming**						
Full-time	Part-time		Full-time	Part-time		
54	11		51	3		0.06
**Daily period at the farm**						
≤ 8 hours	> 8 hours		≤ 8 hours	> 8 hours		
19	46		9	45		0.11
**Family tradition in farming**						
Yes	No		Yes	No		
52	13		51	3		0.021
**Presence of working staff in the flock**						
Yes	No		Yes	No		
22	43		12	42		0.16

Table S8. Results of univariable analysis for associations with optional vaccination against contagious ecthyma in 325 sheep flocks and 119 goat herds in Greece.

(a) Sheep flocks

Vaccination performed (*n* = 3)				Vaccination not performed (*n* = 322)				*p*
**Management system applied in the farm**								
Intensive	Semi-intensive	Semi-extensive	Extensive	Intensive	Semi-intensive	Semi-extensive	Extensive	
1	1	1	0	43	139	115	25	0.76
**No. of ewes in the flock**								
≤ 165 ewes	166-330 ewes	331-500 ewes	> 500 ewes	≤ 165 ewes	166-330 ewes	331-500 ewes	> 500 ewes	
0	1	0	2	88	119	66	49	0.09
**Breed of animals**								
Crossbreeds	Imported breeds	Local breeds		Crossbreeds	Imported breeds	Local breeds		
0	3	0		43	136	143		0.13
**Milking mode in flock**								
Machine-milking	Hand-milking			Machine-milking	Hand-milking			
3	0			252	70			0.36
**Average age of culling ewes**								
≤ 6.5 years	> 6.5 years			≤ 6.5 years	> 6.5 years			
1	2			241	81			0.10
**Start of lambing period**								
All year	Autumn	Winter	Spring - Summer	All year	Autumn	Winter	Spring - Summer	
0	3	0	0	18	242	55	7	
**Collaboration with a veterinarian**								
Yes	No			Yes	No			
3	0			280	42			0.50
**Age of newborns taken away from dam**								
≤ 40 days	41 – 60 days	≥ 61 days		≤ 40 days	41 – 60 days	≥ 61 days		
2	1	0		115	171	36		0.51
**Age of the farmer**								
Up to 50 years	Over 50 years			Up to 50 years	Over 50 years			
2	1			195	127			0.83
**Length of previous animal farming experience of the farmer**								
≤ 5 years	> 5 years			≤ 5 years	> 5 years			
1	2			73	249			0.66

**Education of the farmer**						
Primary education	Secondary or post-secondary education	Tertiary education	Primary education	Secondary or post-secondary education	Tertiary education	
2	1	0	55	224	43	0.08
**Professional involvement in farming**						
Full-time	Part-time		Full-time	Part-time		
3	0		289	33		0.56
**Daily period at the farm**						
≤ 8 hours	> 8 hours		≤ 8 hours	> 8 hours		
2	1		97	225		0.17
**Family tradition in farming**						
Yes	No		Yes	No		
2	1		281	41		0.29
**Presence of working staff in the flock**						
Yes	No		Yes	No		
3	0		120	202		0.026

(b) Goat herds

**Vaccination performed (*n* = 1)**				**Vaccination not performed (*n* = 118)**				***p***
**Management system applied in the farm**								
Intensive	Semi-intensive	Semi-extensive	Extensive	Intensive	Semi-intensive	Semi-extensive	Extensive	
0	0	1	0	9	29	60	20	0.81
**No. of does in the herd**								
≤ 165 ewes	166-330 ewes	331-500 ewes	> 500 ewes	≤ 165 ewes	166-330 ewes	331-500 ewes	> 500 ewes	
1	0	0	0	55	37	13	13	0.77
**Breed of animals**								
Crossbreeds	Imported breeds	Local breeds		Crossbreeds	Imported breeds	Local breeds		
1	0	0		17	45	56		0.06
**Milking mode in herd**								
Machine-milking	Hand-milking			Machine-milking	Hand-milking			
1	0			65	53			0.37
**Average age of culling does**								
≤ 6.5 years	> 6.5 years			≤ 6.5 years	> 6.5 years			
0	1			50	68			0.39

**Start of kidding period**								
All year	Autumn	Winter	Spring - Summer	All year	Autumn	Winter	Spring - Summer	
0	1	0	0	8	62	44	4	0.83
**Collaboration with a veterinarian**								
Yes	No			Yes	No			
1	0			100	18			0.67
**Age of newborns taken away from dam**								
≤ 40 days	41 – 60 days	≥ 61 days		≤ 40 days	41 – 60 days	≥ 61 days		
0	1	0		26	43	49		0.42
**Age of the farmer**								
Up to 50 years	Over 50 years			Up to 50 years	Over 50 years			
0	1			73	45			0.21
**Length of previous animal farming experience of the farmer**								
≤ 5 years	> 5 years			≤ 5 years	> 5 years			
0	1			24	94			0.61
**Education of the farmer**								
Primary education	Secondary or post-secondary education	Tertiary education		Primary education	Secondary or post-secondary education	Tertiary education		
1	0	0		19	89	10		0.08
**Professional involvement in farming**								
Full-time	Part-time			Full-time	Part-time			
1	0			105	13			0.73
**Daily period at the farm**								
≤ 8 hours	> 8 hours			≤ 8 hours	> 8 hours			
1	0			27	91			0.07
**Family tradition in farming**								
Yes	No			Yes	No			
1	0			102	16			0.69
**Presence of working staff in the flock**								
Yes	No			Yes	No			
1	0			33	85			0.11

Table S9. Results of univariable analysis for associations with optional vaccination against foot-rot in 325 sheep flocks in Greece.

**Vaccination performed (*n* = 5)**				**Vaccination not performed (*n* = 320)**				***p***
**Management system applied in the farm**								
Intensive	Semi-intensive	Semi-extensive	Extensive	Intensive	Semi-intensive	Semi-extensive	Extensive	
2	2	1	0	42	138	115	25	0.34
**No. of ewes in the flock**								
≤ 165 ewes	166-330 ewes	331-500 ewes	> 500 ewes	≤ 165 ewes	166-330 ewes	331-500 ewes	> 500 ewes	
1	2	1	1	87	118	65	50	0.98
**Breed of animals**								
Crossbreeds	Imported breeds	Local breeds		Crossbreeds	Imported breeds	Local breeds		
0	4	1		43	135	142		0.23
**Total land available for grazing (acres per animal)**								
≤ 0.50	0.51 – 2.00	≥ 2.00		≤ 0.50	0.51 – 2.00	≥ 2.00		
3	1	1		115	129	76		0.24
**Material of the floor of the barn**								
Soil	Other			Soil	Other			
5	0			285	35			0.43
**Use of straw as bedding material**								
Yes	No			Yes	No			
4	1			264	56			0.88
**Milking mode in flock**								
Machine-milking	Hand-milking			Machine-milking	Hand-milking			
5	0			250	70			0.24
**Average age of culling ewes**								
≤ 6.5 years	> 6.5 years			≤ 6.5 years	> 6.5 years			
5	0			237	83			0.19
**Start of lambing period**								
All year	Autumn	Winter	Spring - Summer	All year	Autumn	Winter	Spring - Summer	
0	5	0	0	18	240	55	7	0.65
**Collaboration with a veterinarian**								
Yes	No			Yes	No			
4	1			279	41			0.63
**Age of newborns taken away from dam**								
≤ 40 days	41 – 60 days	≥ 61 days		≤ 40 days	41 – 60 days	≥ 61 days		
2	2	1		115	170	35		0.76

**Number of daily milking sessions**						
One	Two	Three	One	Two	Three	
0	4	1	1	260	59	0.99
**Age of the farmer**						
Up to 50 years	Over 50 years		Up to 50 years	Over 50 years		
3	2		194	126		0.98
**Length of previous animal farming experience of the farmer**						
≤ 5 years	> 5 years		≤ 5 years	> 5 years		
0	5		74	246		0.22
**Education of the farmer**						
Primary education	Secondary or post-secondary education	Tertiary education	Primary education	Secondary or post-secondary education	Tertiary education	
0	2	3	57	223	40	0.007
**Professional involvement in farming**						
Full-time	Part-time		Full-time	Part-time		
5	0		287	33		0.45
**Daily period at the farm**						
≤ 8 hours	> 8 hours		≤ 8 hours	> 8 hours		
3	2		96	224		0.15
**Family tradition in farming**						
Yes	No		Yes	No		
4	1		279	41		0.63
**Presence of working staff in the flock**						
Yes	No		Yes	No		
2	3		121	199		0.92

Table S10. Results of univariable analysis for associations with optional vaccination against paratuberculosis in 325 sheep flocks and 119 goat herds in Greece.

(a) Sheep flocks

**Vaccination performed (n = 11)**				**Vaccination not performed (n = 314)**				p
**Management system applied in the farm**								
Intensive	Semi-intensive	Semi-extensive	Extensive	Intensive	Semi-intensive	Semi-extensive	Extensive	
1	4	6	0	43	136	110	25	0.51
**No. of ewes in the flock**								
≤ 165 ewes	166-330 ewes	331-500 ewes	> 500 ewes	≤ 165 ewes	166-330 ewes	331-500 ewes	> 500 ewes	
3	5	2	1	86	114	64	50	0.90
**Breed of animals**								
Crossbreeds	Imported breeds	Local breeds		Crossbreeds	Imported breeds	Local breeds		
0	5	6		43	134	137		0.40
**Total land available for grazing (acres per animal)**								
≤ 0.50	0.51 – 2.00	≥ 2.00		≤ 0.50	0.51 – 2.00	≥ 2.00		
2	6	3		116	124	74		0.43
**Material of the floor of the barn**								
Soil	Other			Soil	Other			
11	0			281	33			0.43
**Use of straw as bedding material**								
Yes	No			Yes	No			
10	1			258	56			0.45
**Milking mode in flock**								
Machine-milking	Hand-milking			Machine-milking	Hand-milking			
10	1			176	138			0.022
**Average age of culling ewes**								
≤ 6.5 years	> 6.5 years			≤ 6.5 years	> 6.5 years			
6	5			236	78			0.12
**Start of lambing period**								
All year	Autumn	Winter	Spring - Summer	All year	Autumn	Winter	Spring - Summer	
0	6	5	0	18	239	50	7	0.07
**Collaboration with a veterinarian**								
Yes	No			Yes	No			
9	2			274	40			0.60

**Age of newborns taken away from dam**						
≤ 40 days	41 – 60 days	≥ 61 days	≤ 40 days	41 – 60 days	≥ 61 days	
2	9	0	115	163	36	0.13
**Number of daily milking sessions**						
One	Two	Three	One	Two	Three	
0	8	3	1	256	57	0.74
**Age of the farmer**						
Up to 50 years	Over 50 years		Up to 50 years	Over 50 years		
8	3		189	125		0.40
**Length of previous animal farming experience of the farmer**						
≤ 5 years	> 5 years		≤ 5 years	> 5 years		
1	10		73	241		0.27
**Education of the farmer**						
Primary education	Secondary or post-secondary education	Tertiary education	Primary education	Secondary or post-secondary education	Tertiary education	
2	7	2	55	218	41	0.88
**Professional involvement in farming**						
Full-time	Part-time		Full-time	Part-time		
10	1		282	32		0.91
**Daily period at the farm**						
≤ 8 hours	> 8 hours		≤ 8 hours	> 8 hours		
3	8		96	218		0.82
**Family tradition in farming**						
Yes	No		Yes	No		
11	0		272	42		0.19
**Presence of working staff in the flock**						
Yes	No		Yes	No		
3	8		194	120		0.021

(b) Goat herds

**Vaccination performed (n = 31)**				**Vaccination not performed (n = 88)**				***p***
**Management system applied in the farm**								
Intensive	Semi-intensive	Semi-extensive	Extensive	Intensive	Semi-intensive	Semi-extensive	Extensive	
2	9	13	7	7	20	48	13	0.58

**No. of does in the herd**								
≤ 165 does	166-330 does	331-500 does	> 500 does	≤ 165 does	166-330 does	331-500 does	> 500 does	
8	11	5	7	48	26	8	6	0.015
**Breed of animals**								
Crossbreeds	Imported breeds	Local breeds	Crossbreeds	Imported breeds	Local breeds			
4	12	15	14	33	41			0.92
**Total land available for grazing (acres per animal)**								
≤ 0.50	0.51 – 2.00	≥ 2.00	≤ 0.50	0.51 – 2.00	≥ 2.00			
4	12	15	13	19	56			0.17
**Material of the floor of the barn**								
Soil	Other	Soil	Other					
25	6	80	8					0.13
**Use of straw as bedding material**								
Yes	No	Yes	No					
19	12	57	31					0.73
**Milking mode in herd**								
Machine-milking	Hand-milking	Machine-milking	Hand-milking					
19	12	47	41					0.45
**Average age of culling does**								
≤ 6.5 years	> 6.5 years	≤ 6.5 years	> 6.5 years					
13	18	37	51					0.99
**Start of kidding period**								
All year	Autumn	Winter	Spring - Summer	All year	Autumn	Winter	Spring - Summer	
2	14	14	1	6	49	30	3	0.74
**Collaboration with a veterinarian**								
Yes	No	Yes	No					
26	5	62	26					0.14
**Age of newborns taken away from dam**								
≤ 40 days	41 – 60 days	≥ 61 days	≤ 40 days	41 – 60 days	≥ 61 days			
7	9	15	19	35	34			0.53
**Number of daily milking sessions**								
One	Two	Three	One	Two	Three			
0	31	0	4	77	7			0.12

**Age of the farmer**						
Up to 50 years	Over 50 years	Up to 50 years	Over 50 years			
21	10	52	36			0.38
**Length of previous animal farming experience of the farmer**						
≤ 5 years	> 5 years	≤ 5 years	> 5 years			
5	26	19	69			0.51
**Education of the farmer**						
Primary education	Secondary or post-secondary education	Tertiary education	Primary education	Secondary or post-secondary education	Tertiary education	
4	24	3	16	65	7	0.78
**Professional involvement in farming**						
Full-time	Part-time	Full-time	Part-time			
26	5	79	9			0.38
**Daily period at the farm**						
≤ 8 hours	> 8 hours	≤ 8 hours	> 8 hours			
5	26	23	65			0.26
**Family tradition in farming**						
Yes	No	Yes	No			
29	2	74	14			0.18
**Presence of working staff in the flock**						
Yes	No	Yes	No			
10	21	24	64			0.60

Table S11. Results of univariable analysis for associations with optional vaccination against pneumonia in 325 sheep flocks and 119 goat herds in Greece.

(a) Sheep flocks

Management system applied in the farm

Vaccination performed (*n* = 144)				Vaccination not performed (*n* = 181)				*p*
Intensive	Semi-intensive	Semi-extensive	Extensive	Intensive	Semi-intensive	Semi-extensive	Extensive	
23	59	54	8	21	81	62	17	0.37

No. of ewes in the flock

Vaccination performed (*n* = 144)				Vaccination not performed (*n* = 181)				*p*
≤ 165 ewes	166-330 ewes	331-500 ewes	> 500 ewes	≤ 165 ewes	166-330 ewes	331-500 ewes	> 500 ewes	
36	54	26	28	52	66	40	23	0.33

Breed of animals

Vaccination performed (*n* = 144)			Vaccination not performed (*n* = 181)			*p*
Crossbreeds	Imported breeds	Local breeds	Crossbreeds	Imported breeds	Local breeds	
19	64	61	24	75	82	0.85

Total land available for grazing (acres per animal)

Vaccination performed (*n* = 144)			Vaccination not performed (*n* = 181)			*p*
≤ 0.50	0.51 – 2.00	≥ 2.00	≤ 0.50	0.51 – 2.00	≥ 2.00	
51	69	24	67	61	53	0.009

Milking mode in flock

Vaccination performed (*n* = 144)		Vaccination not performed (*n* = 181)		*p*
Machine-milking	Hand-milking	Machine-milking	Hand-milking	
116	28	139	42	0.41

Average age of culling ewes

Vaccination performed (*n* = 144)		Vaccination not performed (*n* = 181)		*p*
≤ 6.5 years	> 6.5 years	≤ 6.5 years	> 6.5 years	
112	32	130	51	0.22

Start of lambing period

Vaccination performed (*n* = 144)				Vaccination not performed (*n* = 181)				*p*
All year	Autumn	Winter	Spring - Summer	All year	Autumn	Winter	Spring - Summer	
7	107	24	6	11	138	31	1	0.16

Collaboration with a veterinarian

Vaccination performed (*n* = 144)		Vaccination not performed (*n* = 181)		*p*
Yes	No	Yes	No	
129	15	154	27	0.23

Age of newborns taken away from dam

Vaccination performed (*n* = 144)			Vaccination not performed (*n* = 181)			*p*
≤ 40 days	41 – 60 days	≥ 61 days	≤ 40 days	41 – 60 days	≥ 61 days	
57	73	14	60	99	22	0.45

Routine administration of antibiotics to newborns

Vaccination performed (*n* = 144)		Vaccination not performed (*n* = 181)		*p*
Yes	No	Yes	No	
41	103	29	152	0.007

**Number of daily milking sessions**						
One	Two	Three	One	Two	Three	
1	111	32	0	153	28	0.15
**Age of the farmer**						
Up to 50 years	Over 50 years		Up to 50 years	Over 50 years		
87	57		110	71		0.95
**Length of previous animal farming experience of the farmer**						
≤ 5 years	> 5 years		≤ 5 years	> 5 years		
31	113		43	138		0.63
**Education of the farmer**						
Primary education	Secondary or post-secondary education	Tertiary education	Primary education	Secondary or post-secondary education	Tertiary education	
26	97	21	31	128	22	0.77
**Professional involvement in farming**						
Full-time	Part-time		Full-time	Part-time		
129	15		163	18		0.89
**Daily period at the farm**						
≤ 8 hours	> 8 hours		≤ 8 hours	> 8 hours		
34	110		65	116		0.017
**Family tradition in farming**						
Yes	No		Yes	No		
125	19		158	23		0.90
**Presence of working staff in the flock**						
Yes	No		Yes	No		
59	85		64	117		0.30

(b) Goat herds

**Vaccination performed (n = 39)**				**Vaccination not performed (n = 80)**				p
**Management system applied in the farm**								
Intensive	Semi-intensive	Semi-extensive	Extensive	Intensive	Semi-intensive	Semi-extensive	Extensive	
4	8	21	6	5	21	40	14	0.78
**No. of does in the herd**								
≤ 165 does	166-330 does	331-500 does	> 500 does	≤ 165 does	166-330 does	331-500 does	> 500 does	
17	13	8	1	39	24	5	12	0.032

**Breed of animals**								
Crossbreeds	Imported breeds	Local breeds	Crossbreeds	Imported breeds	Local breeds			
5	17	17	13	28	39			0.65
**Total land available for grazing (acres per animal)**								
≤ 0.50	0.51 – 2.00	≥ 2.00	≤ 0.50	0.51 – 2.00	≥ 2.00			
7	9	23	10	22	48			0.69
**Milking mode in herd**								
Machine-milking	Hand-milking	Machine-milking	Hand-milking					
22	17	44	36					0.88
**Average age of culling does**								
≤ 6.5 years	> 6.5 years	≤ 6.5 years	> 6.5 years					
16	23	34	46					0.88
**Start of kidding period**								
All year	Autumn	Winter	Spring - Summer	All year	Autumn	Winter	Spring - Summer	
3	20	14	2	5	43	30	2	0.88
**Collaboration with a veterinarian**								
Yes	No	Yes	No					
33	6	68	12					0.96
**Age of newborns taken away from dam**								
≤ 40 days	41 – 60 days	≥ 61 days	≤ 40 days	41 – 60 days	≥ 61 days			
7	15	17	19	29	32			0.77
**Routine administration of antibiotics to newborns**								
Yes	No	Yes	No					
8	31	22	58					0.41
**Number of daily milking sessions**								
One	Two	Three	One	Two	Three			
2	34	3	2	74	4			0.62
**Age of the farmer**								
Up to 50 years	Over 50 years	Up to 50 years	Over 50 years					
23	16	50	30					0.71
**Length of previous animal farming experience of the farmer**								
≤ 5 years	> 5 years	≤ 5 years	> 5 years					
6	33	18	52					0.21

**Education of the farmer**						
Primary education	Secondary or post-secondary education	Tertiary education	Primary education	Secondary or post-secondary education	Tertiary education	
5	32	2	15	57	8	0.43

**Professional involvement in farming**				
Full-time	Part-time	Full-time	Part-time	
34	5	71	9	0.80
**Daily period at the farm**				
≤ 8 hours	> 8 hours	≤ 8 hours	> 8 hours	
7	32	21	59	0.32
**Family tradition in farming**				
Yes	No	Yes	No	
36	3	67	13	0.21
**Presence of working staff in the flock**				
Yes	No	Yes	No	
15	24	19	61	0.10

Table S12. Results of univariable analysis for associations with optional vaccination against staphylococcal mastitis in 325 sheep flocks and 119 goat herds in Greece.

(a) Sheep flocks

**Vaccination performed (*n* = 126)**				**Vaccination not performed (*n* = 199)**				***p***
**Management system applied in the farm**								
Intensive	Semi-intensive	Semi-extensive	Extensive	Intensive	Semi-intensive	Semi-extensive	Extensive	
20	66	36	4	24	74	80	21	0.004
**No. of ewes in the flock**								
≤ 165 ewes	166-330 ewes	331-500 ewes	> 500 ewes	≤ 165 ewes	166-330 ewes	331-500 ewes	> 500 ewes	
39	48	24	15	49	72	42	36	0.35
**Breed of animals**								
Crossbreeds	Imported breeds	Local breeds		Crossbreeds	Imported breeds	Local breeds		
15	66	45		28	73	98		0.019
**Total land available for grazing (acres per animal)**								
≤ 0.50	0.51 – 2.00	≥ 2.00		≤ 0.50	0.51 – 2.00	≥ 2.00		
52	50	24		66	80	53		0.19
**Milking mode in flock**								
Machine-milking	Hand-milking			Machine-milking	Hand-milking			
109	17			146	53			0.005
**Average age of culling ewes**								
≤ 6.5 years	> 6.5 years			≤ 6.5 years	> 6.5 years			
100	26			142	57			0.11
**Start of lambing period**								
All year	Autumn	Winter	Spring - Summer	All year	Autumn	Winter	Spring - Summer	
6	102	14	4	12	143	41	3	0.10
**Collaboration with a veterinarian**								
Yes	No			Yes	No			
119	7			164	35			0.009
**Age of newborns taken away from dam**								
≤ 40 days	41 – 60 days	≥ 61 days		≤ 40 days	41 – 60 days	≥ 61 days		
51	68	7		66	104	29		0.033
**Number of daily milking sessions**								
One	Two	Three		One	Two	Three		
0	95	31		1	169	29		0.06

**Use of laboratory diagnostic examinations in samples of milk**						
Yes	No		Yes	No		
39	87		31	168		0.001
**Application of teat disinfection during milking**						
Yes	No		Yes	No		
27	99		25	174		0.033
**Application of 'dry-period treatment'**						
Yes	No		Yes	No		
30	96		23	176		0.004
**Duration of dry-period (months)**						
≤ 2	> 2		≤ 2	> 2		
48	78		59	140		0.11
**Age of the farmer**						
Up to 50 years	Over 50 years		Up to 50 years	Over 50 years		
85	41		112	87		0.044
**Length of previous animal farming experience of the farmer**						
≤ 5 years	> 5 years		≤ 5 years	> 5 years		
37	89		37	162		0.024
**Education of the farmer**						
Primary education	Secondary or post-secondary education	Tertiary education	Primary education	Secondary or post-secondary education	Tertiary education	
25	83	18	32	142	25	0.57
**Professional involvement in farming**						
Full-time	Part-time		Full-time	Part-time		
109	17		183	16		0.11
**Daily period at the farm**						
≤ 8 hours	> 8 hours		≤ 8 hours	> 8 hours		
38	88		61	138		0.92
**Family tradition in farming**						
Yes	No		Yes	No		
110	16		173	26		0.92
**Presence of working staff in the flock**						
Yes	No		Yes	No		
52	74		71	128		0.31

(b) Goat herds

Vaccination performed (n = 34)				Vaccination not performed (n = 85)				p
**Management system applied in the farm**								
Intensive	Semi-intensive	Semi-extensive	Extensive	Intensive	Semi-intensive	Semi-extensive	Extensive	
2	13	19	0	7	16	42	20	0.007
**No. of does in the herd**								
≤ 165 ewes	166-330 ewes	331-500 ewes	> 500 ewes	≤ 165 ewes	166-330 ewes	331-500 ewes	> 500 ewes	
17	11	5	1	39	26	8	12	0.32
**Breed of animals**								
Crossbreeds	Imported breeds	Local breeds		Crossbreeds	Imported breeds	Local breeds		
6	17	11		12	28	45		0.12
**Total land available for grazing (acres per animal)**								
≤ 0.50	0.51 – 2.00	≥ 2.00		≤ 0.50	0.51 – 2.00	≥ 2.00		
5	8	21		12	23	50		0.92
**Milking mode in herd**								
Machine-milking	Hand-milking			Machine-milking	Hand-milking			
27	7			39	46			0.0009
**Average age of culling does**								
≤ 6.5 years	> 6.5 years			≤ 6.5 years	> 6.5 years			
18	16			32	53			0.13
**Start of kidding period**								
All year	Autumn	Winter	Spring - Summer	All year	Autumn	Winter	Spring - Summer	
3	20	10	1	5	43	34	3	0.71
**Collaboration with a veterinarian**								
Yes	No			Yes	No			
31	3			70	15			0.22
**Age of newborns taken away from dam**								
≤ 40 days	41 – 60 days	≥ 61 days		≤ 40 days	41 – 60 days	≥ 61 days		
12	14	8		14	30	41		0.021
**Number of daily milking sessions**								
One	Two	Three		One	Two	Three		
0	29	5		4	79	2		0.018
**Use of laboratory diagnostic examinations in samples of milk**								
Yes	No			Yes	No			
24	10			70	15			0.15

<table>
<tr><td colspan="12">Application of teat disinfection during milking</td><td></td></tr>
<tr><td colspan="3">Yes</td><td colspan="3">No</td><td colspan="3">Yes</td><td colspan="3">No</td><td></td></tr>
<tr><td colspan="3">7</td><td colspan="3">27</td><td colspan="3">5</td><td colspan="3">80</td><td>0.017</td></tr>
<tr><td colspan="12">Application of ‘dry-period treatment’</td><td></td></tr>
<tr><td colspan="3">Yes</td><td colspan="3">No</td><td colspan="3">Yes</td><td colspan="3">No</td><td></td></tr>
<tr><td colspan="3">8</td><td colspan="3">26</td><td colspan="3">7</td><td colspan="3">78</td><td>0.023</td></tr>
<tr><td colspan="12">Duration of dry-period (months)</td><td></td></tr>
<tr><td colspan="3">≤ 2</td><td colspan="3">> 2</td><td colspan="3">≤ 2</td><td colspan="3">> 2</td><td></td></tr>
<tr><td colspan="3">11</td><td colspan="3">23</td><td colspan="3">28</td><td colspan="3">57</td><td>0.95</td></tr>
<tr><td colspan="12">Age of the farmer</td><td></td></tr>
<tr><td colspan="3">Up to 50 years</td><td colspan="3">Over 50 years</td><td colspan="3">Up to 50 years</td><td colspan="3">Over 50 years</td><td></td></tr>
<tr><td colspan="3">23</td><td colspan="3">11</td><td colspan="3">50</td><td colspan="3">35</td><td>0.37</td></tr>
<tr><td colspan="12">Length of previous animal farming experience of the farmer</td><td></td></tr>
<tr><td colspan="3">≤ 5 years</td><td colspan="3">> 5 years</td><td colspan="3">≤ 5 years</td><td colspan="3">> 5 years</td><td></td></tr>
<tr><td colspan="3">7</td><td colspan="3">27</td><td colspan="3">17</td><td colspan="3">68</td><td>0.94</td></tr>
<tr><td colspan="12">Education of the farmer</td><td></td></tr>
<tr><td colspan="2">Primary education</td><td colspan="2">Secondary or post-secondary education</td><td colspan="2">Tertiary education</td><td colspan="2">Primary education</td><td colspan="2">Secondary or post-secondary education</td><td colspan="2">Tertiary education</td><td></td></tr>
<tr><td colspan="2">4</td><td colspan="2">27</td><td colspan="2">3</td><td colspan="2">16</td><td colspan="2">62</td><td colspan="2">7</td><td>0.65</td></tr>
<tr><td colspan="12">Professional involvement in farming</td><td></td></tr>
<tr><td colspan="3">Full-time</td><td colspan="3">Part-time</td><td colspan="3">Full-time</td><td colspan="3">Part-time</td><td></td></tr>
<tr><td colspan="3">30</td><td colspan="3">4</td><td colspan="3">75</td><td colspan="3">10</td><td>1.00</td></tr>
<tr><td colspan="12">Daily period at the farm</td><td></td></tr>
<tr><td colspan="3">≤ 8 hours</td><td colspan="3">> 8 hours</td><td colspan="3">≤ 8 hours</td><td colspan="3">> 8 hours</td><td></td></tr>
<tr><td colspan="3">5</td><td colspan="3">29</td><td colspan="3">23</td><td colspan="3">62</td><td>0.15</td></tr>
<tr><td colspan="12">Family tradition in farming</td><td></td></tr>
<tr><td colspan="3">Yes</td><td colspan="3">No</td><td colspan="3">Yes</td><td colspan="3">No</td><td></td></tr>
<tr><td colspan="3">29</td><td colspan="3">5</td><td colspan="3">74</td><td colspan="3">11</td><td>0.80</td></tr>
<tr><td colspan="12">Presence of working staff in the flock</td><td></td></tr>
<tr><td colspan="3">Yes</td><td colspan="3">No</td><td colspan="3">Yes</td><td colspan="3">No</td><td></td></tr>
<tr><td colspan="3">13</td><td colspan="3">21</td><td colspan="3">21</td><td colspan="3">64</td><td>0.14</td></tr>
</table>

Table S13. Results of univariable analysis for associations with total number of optional vaccinations in 325 sheep flocks and 119 goat herds in Greece.

(a) Sheep flocks

				p
**Management system applied in the farm**				
Intensive	Semi-intensive	Semi-extensive	Extensive	
3.43 ± 0.17	3.04 ± 0.10	2.62 ± 0.12	1.72 ± 0.26	< 0.0001
**No. of ewes in the flock**				
r = 0.055				0.16
**Total land available for grazing (acres per animal)**				
r = –0.097				0.04
**Milking mode in flock**				
Machine-milking		Hand-milking		
3.04 ± 0.08		2.14 ± 0.15		< 0.0001
**Average age of culling ewes**				
r = –0.162				0.002
**Start of lambing period**				
All year	Autumn	Winter	Spring - Summer	
2.72 ± 0.29	2.90 ± 0.08	2.58 ± 0.19	3.28 ± 0.42	0.32
**Collaboration with a veterinarian**				
Yes		No		
2.94 ± 0.08		2.17 ± 0.19		0.0004
**Age of newborns taken away from dam**				
r = –0.281				< 0.0001
**Routine administration of antibiotics to newborns**				
Yes		No		
3.33 ± 0.14		2.71 ± 0.08		0.0005
**Number of daily milking sessions**				
r = –0.214				< 0.0001
**Use of laboratory diagnostic examinations in samples of milk**				
Yes		No		
3.43 ± 0.16		2.68 ± 0.08		< 0.0001
**Application of 'dry-period treatment'**				
Yes		No		
3.33 ± 0.18		2.71 ± 0.08		0.0005

	**Duration of dry-period (months)**		
	$r = -0.176$		0.007
	**Age of the farmer**		
	$r = -0.250$		0.007
	**Education of the farmer**		
Primary education	Secondary or post-secondary education	Tertiary education	
3.02 ± 0.08	2.77 ± 0.07	2.98 ± 0.22	0.36
	**Professional involvement in farming**		
Full-time		Part-time	
2.82 ± 0.16		3.06 ± 0.21	0.32
	**Daily period at the farm**		
	$r = -0.002$		0.49
	**Family tradition in farming**		
Yes		No	
2.83 ± 0.08		2.95 ± 0.22	0.57
	**Presence of working staff in the flock**		
Yes		No	
3.13 ± 0.11		2.67 ± 0.09	0.002

(b) Goat herds

			p
	**No. of does in the herd**		
	$r = 0.090$		0.49
	**Breed of animals**		
Crossbreeds	Imported breeds	Local breeds	
3.11 ± 0.29	3.13 ± 0.20	2.36 ± 0.18	0.008
	**Material of the floor of the barn**		
Soil		Other	
2.76 ± 0.13		2.80 ± 0.39	0.93
	**Milking mode in flock**		
Machine-milking		Hand-milking	
3.09 ± 0.16		2.36 ± 0.18	0.003
	**Average age of culling ewes**		
	$r = 0.199$		0.015

**Collaboration with a veterinarian**			
Yes		No	
2.87 ± 0.14		2.17 ± 0.23	0.043
**Age of newborns taken away from dam**			
	$r = -0.174$		0.029
**Routine administration of antibiotics to newborns**			
Yes		No	
2.80 ± 0.23		2.75 ± 0.15	0.87
**Number of daily milking sessions**			
	$r = 0.219$		0.008
**Use of laboratory diagnostic examinations in samples of milk**			
Yes		No	
3.48 ± 0.27		2.57 ± 0.14	0.003
**Application of 'dry-period treatment'**			
Yes		No	
3.67 ± 0.30		2.63 ± 0.26	0.006
**Education of the farmer**			
Primary education	Secondary or post-secondary education	Tertiary education	
2.40 ± 0.32	2.84 ± 0.34	2.80 ± 0.44	0.42
**Professional involvement in farming**			
Full-time		Part-time	
2.73 ± 0.14		3.00 ± 0.30	0.49
**Daily period at the farm**			
	$r = 0.093$		0.16
**Family tradition in farming**			
Yes		No	
2.77 ± 0.13		2.75 ± 0.13	0.96
**Presence of working staff in the flock**			
Yes		No	
3.12 ± 0.25		2.62 ± 0.14	0.07
